# Multi-walled carbon nanotube induces nitrative DNA damage in human lung epithelial cells via HMGB1-RAGE interaction and Toll-like receptor 9 activation

**DOI:** 10.1186/s12989-016-0127-7

**Published:** 2016-03-29

**Authors:** Yusuke Hiraku, Feiye Guo, Ning Ma, Tatsuhiko Yamada, Shumin Wang, Shosuke Kawanishi, Mariko Murata

**Affiliations:** 1 Department of Environmental and Molecular Medicine, Mie University Graduate School of Medicine, 2-174 Edobashi, Tsu, Mie 514-8507 Japan; 2 Faculty of Nursing Science, Suzuka University of Medical Science, 3500-3 Minami-Tamagaki-cho, Suzuka, Mie 513-8670 Japan; 3 Faculty of Pharmaceutical Sciences, Suzuka University of Medical Science, 3500-3 Minami-Tamagaki-cho, Suzuka, Mie 513-8670 Japan

**Keywords:** Carbon nanotube, Endocytosis, Cytotoxicity, High-mobility group box-1, Receptor for advanced glycation end-products, Toll-like receptor, Nitric oxide, DNA damage, 8-nitroguanine, Carcinogenesis

## Abstract

**Background:**

Carbon nanotube (CNT) is used for various industrial purposes, but exhibits carcinogenic effects in experimental animals. Chronic inflammation in the respiratory system may participate in CNT-induced carcinogenesis. 8-Nitroguanine (8-nitroG) is a mutagenic DNA lesion formed during inflammation. We have previously reported that multi-walled CNT (MWCNT) induced 8-nitroG formation in lung epithelial cells and this process involved endocytosis. To clarify the mechanism of CNT-induced carcinogenesis, we examined the role of Toll-like receptor (TLR) 9, which resides in endosomes and lysosomes, in 8-nitroG formation in human lung epithelial cell lines.

**Methods:**

We performed immunocytochemistry to examine 8-nitroG formation in A549 and HBEpC cells treated with MWCNT with a length of 1-2 μm (CNT-S) or 5-15 μm (CNT-L) and a diameter of 20-40 nm. We examined inhibitory effects of endocytosis inhibitors, small interfering RNA (siRNA) for TLR9, and antibodies against high-mobility group box-1 (HMGB1) and receptor for advanced glycation end-products (RAGE) on 8-nitroG formation. The release of HMGB1 and double-stranded DNA (dsDNA) into the culture supernatant from MWCNT-treated cells was examined by ELISA and fluorometric analysis, respectively. The association of these molecules was examined by double immunofluorescent staining and co-immunoprecipitation.

**Results:**

CNT-L significantly increased 8-nitroG formation at 0.05 μg/ml in A549 cells and its intensity reached a maximum at 1 μg/ml. CNT-L tended to induce stronger cytotoxicity and 8-nitroG formation than CNT-S. Endocytosis inhibitors, TLR9 siRNA and antibodies against HMGB1 and RAGE largely reduced MWCNT-induced 8-nitroG formation. MWCNT increased the release of HMGB1 and dsDNA from A549 cells into culture supernatant. The culture supernatant of MWCNT-exposed cells induced 8-nitroG formation in fresh A549 cells. Double immunofluorescent staining and co-immunoprecipitation showed that TLR9 was associated with HMGB1 and RAGE in lysosomes of MWCNT-treated cells.

**Conclusions:**

MWCNT induces injury or necrosis of lung epithelial cells, which release HMGB1 and DNA into the extracellular space. The HMGB1-DNA complex binds to RAGE on neighboring cells and then CpG DNA is recognized by TLR9 in lysosomes, leading to generation of nitric oxide and 8-nitroG formation. This is the first study demonstrating that TLR9 and related molecules participate in MWCNT-induced genotoxicity and may contribute to carcinogenesis.

**Electronic supplementary material:**

The online version of this article (doi:10.1186/s12989-016-0127-7) contains supplementary material, which is available to authorized users.

## Background

Carbon nanotube (CNT) is a promising nanomaterial that has been used for various industrial and medical purposes, because of its unique physicochemical properties, such as high electrical conductivity and excellent strength [1, 2]. However, its fibrous-like shape and durability has raised a concern that the toxicity and carcinogenicity may be analogous to those observed with asbestos [3–5]. Animal studies have demonstrated that intraperitoneal [5–7] and intrascrotal [8] administration of CNT induced mesothelioma. CNT causes length-dependent pathohistological changes and inflammatory responses in the lungs and pleural cavity of mice [5, 9]. However, the precise mechanism of CNT-induced carcinogenesis remains to be clarified.

Chronic inflammation is considered to play a critical role in carcinogenesis [10–12]. Administration of CNT induces inflammatory responses in lung tissues of experimental animals [13–15]. Under the inflammatory conditions, reactive oxygen (ROS) and nitrogen species (RNS) are generated in inflammatory and epithelial cells and cause DNA damage [16, 17]. 8-Nitroguanine (8-nitroG) is a mutagenic DNA lesion produced by the reaction of guanine with peroxynitrite (ONOO^-^), which is formed from nitric oxide (NO) and superoxide (O_2_^-^) generated under inflammatory conditions [18]. We have demonstrated that 8-nitroG is formed at the sites of carcinogenesis in various animal models and clinical specimens of cancer-prone inflammatory diseases [19–22], including asbestos exposure [23, 24]. We have reported that multi-walled CNT (MWCNT) induced 8-nitroG formation in a lung epithelial cell line and this process involves endocytosis [25]. This finding led us to an idea that endosomal and/or lysosomal molecules may contribute to CNT-induced inflammation and DNA damage.

Toll-like receptors (TLRs) are major contributors to the innate immune system and mediate inflammatory responses against infectious and noninfectious pathogens by recognizing structures conserved among microbial species (pathogen-associated molecular patterns) and endogenous molecules released from damaged cells (damage - associated molecular patterns). Whereas many TLRs are located in the plasma membrane, TLRs 3, 7 and 9 reside on the endosomal and lysosomal membranes [26]. TLR9 recognizes CpG DNA of microorganisms and endogenous origins to mediate inflammatory responses [26, 27]. TLR9 is most abundantly expressed in the spleen of mice [28], and its role in inflammatory responses has well been investigated in immune cells [26]. TLR9 is also expressed in bronchial epithelium and alveolar septa in intact lung tissues of humans [29]. TLR9 is highly expressed in human lung cancer specimens [30], raising the possibility that this receptor contributes to carcinogenesis.

High-mobility group protein B1 (HMGB1) is a nuclear protein released from damaged or necrotic cells and interacts with DNA to form the HMGB1-DNA complex [31]. This complex binds to the receptor for advanced glycation end products (RAGE) on the cell membrane and then activates TLR9-mediated inflammatory responses [31, 32]. RAGE is a multiligand transmembrane receptor constitutively expressed in the lung throughout the life [33]. HMGB1-mediated RAGE activation participates in various pathological conditions including cancer, sepsis, diabetes and Alzheimer’s disease [33]. HMGB1 contributes to asbestos-induced inflammatory responses in cultured cells and experimental animals, and its serum level in asbestos-exposed individuals was significantly higher than that in unexposed subjects [34]. However, the role of TLR9 and related molecules in CNT-induced inflammatory responses and genotoxicity has not been investigated.

In this study, we examined 8-nitroG formation in MWCNT-treated A549 human alveolar epithelial cells and HBEpC normal human bronchial epithelial cells. We used two types of MWCNTs with different fiber length to examine the effects of their sizes on 8-nitroG formation, since long CNT fibers tended to cause more prominent inflammatory responses in respiratory systems of experimental animals [5, 9]. To clarify the mechanism of MWCNT-induced DNA damage, we investigated the involvement of the HMGB1-RAGE interaction and TLR9 activation in this process by using antibodies and small interfering RNA (siRNA), respectively. The association of these molecules in MWCNT-treated cells was examined by double immunofluorescent technique and co-immunoprecipitaion.

## Methods

### Preparation of MWCNT

MWCNTs with lengths of 1-2 μm (CNT-S) and 5-15 μm (CNT-L) and diameter of 20-40 nm were obtained from Nanostructured and Amorphous Materials, Inc. (purity, 95-98 %, Houston, TX, USA). Trace metals contained in these MWCNTs were quantified by inductively coupled plasma-atomic emission spectrometry (ICP-AES, ICPS-8100, Shimadzu, Kyoto, Japan) and inductively coupled plasma mass spectrometry (ICP-MS, Agilent7700x, Agilent Technologies, Santa Clara, CA, USA). MWCNT was suspended in Dulbecco’s modified Eagle’s medium (DMEM, Gibco, New York, NY, USA) supplemented with 5 % (v/v) heat-inactivated fetal bovine serum (FBS, Cambrex Bio Science Walkersville, Walkersville, MD, USA or Biowest, Nuaillé, France) and 100 mg/l kanamycin and vortexed for 1 min. In certain experiments, MWCNT was suspended in phosphate-buffered saline (PBS, pH 7.4) or dispersion medium (PBS supplemented with 5.5 mM D-glucose, 0.6 mg/ml mouse serum albumin, and 0.01 mg/ml 1,2-dipalmitoyl-*sn*-glycero-3-phosphocholine) [13]. Then the suspensions were sonicated for 20 min at 40 W using an ultrasonic homogenizer (Advanced Sonifier Model 450, Branson Ultrasonics, Danbury, CT, USA) to disperse agglomerates. The suspensions containing dispersed MWCNT were stored at –80 °C until use. We thawed and vortexed the suspensions, and then used them for further experiments. Size distribution of MWCNT agglomerates was measured with a Zetasizer Nano particle size analyzer (Malvern, Worcestershire, UK) under the same conditions as those used in experiments.

### MTT assay

To evaluate a cytotoxic effect of MWCNT, 3-(4,5-dimethylthiazol-2-yl)-2,5-diphenyltetrazolium bromide (MTT) assay was performed as reported previously [25]. A549 cells (1 × 10^4^ cells/well, RIKEN BioResource Center, Tsukuba, Japan) were cultured in a 96-well plate overnight and treated with MWCNT for 24 h at 37 °C in DMEM containing 5 % (v/v) FBS and 100 mg/l kanamycin. The culture supernatant was removed and the cells were incubated with 0.5 mg/ml MTT for 4 h at 37 °C, followed by the treatment with dimethylsulfoxide for 10 min at room temperature. The absorbance of each well was measured at 570 nm using a Model 680 microplate reader (Bio-Rad Laboratories, Hercules, CA, USA). To examine of the absorption of MWCNT, we measured UV-visible spectra of the reaction mixture containing 1 μg/ml MWCNT with a UV–visible spectrometer (UV-2500PC, Shimadzu). To examine adsorption effects of MWCNT, we compared the spectra of the reaction mixture containing 0.05 mg/ml MTT before and immediately (0 min) and 20 min after the addition of 1 μg/ml MWCNT.

### Fluorescent immunocytochemistry

Fluorescent immunohistochemistry was performed as described previously [25]. A549 cells (2 × 10^5^ cells/ml) were cultured in DMEM containing 5 % (v/v) FBS and 100 mg/l kanamycin overnight on culture slides (BD Falcon, Franklin Lakes, NJ, USA). HBEpC cells (5 × 10^4^ cells/ml, Cell Applications, San Diego, CA, USA) were cultured in Airway epithelial cell growth medium (PromoCell, Heidelberg, Germany) on culture slides. Then these cells were treated with MWCNT for indicated durations at 37 °C.

To examine the roles of inducible NO synthase (iNOS) and endocytosis in MWCNT-induced 8-nitroG formation, 1 μM 1400 W, 10 μM Bay11-7082 (Bay), 2 mM methyl-β-cyclodextrin (MBCD), 50 μM monodansylcadaverine (MDC) or 1 μM cytochalasin D (CytoD) was added before MWCNT treatment. These inhibitors were purchased from Sigma-Aldrich (St. Louis, MO, USA). We employed these concentrations of the inhibitors, because they did not show significant cytotoxic effects (as shown below) and effectively reduced MWCNT-induced 8-nitroG formation and iNOS expression in our previous study [25]. To examine the role of HMGB1 and RAGE in 8-nitroG formation, the cells were pretreated with 10 μg/mL anti-HMGB1 (raised from full length protein including RAGE-interacting motif, ab77302, Abcam, Cambridge, UK) or anti-RAGE (raised from full length protein including HMGB1-interacting V domain, ab54741, Abcam) mouse monoclonal antibody for 30 min before MWCNT treatment. We employed this concentration of these antibodies according to a previous study [32]. To confirm the specificity of these antibodies, the corresponding isotype control IgG [mouse IgG_1_ (ab18447, Abcam) for anti-HMGB1 antibody and IgG_2a_ (ab18414, Abcam) for anti-RAGE antibody] were used instead. These antibodies were solved in PBS and did not contain any preservatives, such as sodium azide. We confirmed that these antibodies and control IgGs did not show significant cytotoxicity (as shown below). To examine the role of TLR2 and TLR4 in 8-nitroG formation, the cells were pretreated with anti-TLR2 (T2.5, ab185793, Abcam) or anti-TLR4 (HTA125, ab171260, Abcam) mouse monoclonal antibody for 30 min before MWCNT treatment.

After the treatment with MWCNT, the cells were fixed with 4 % (v/v) formaldehyde in PBS for 10 min and treated with 0.5 % (v/v) Triton X100 for 3 min at room temperature. The cells were incubated with 1 % (w/v) skim milk for 1 h at room temperature. To detect 8-nitroG, the cells were incubated with rabbit polyclonal antibody (1 μg/ml) produced by our group [35, 36] overnight at room temperature. To detect iNOS and 8-oxo-7,8-dihydro-2'-deoxyguanosine (8-oxodG), an oxidative DNA lesion, rabbit polyclonal (1:1000, Calbiochem, Darmstadt, Germany) and mouse monoclonal (0.5 μg/ml, Japan Institute for the Control of Aging, Fukuroi, Japan) antibodies were used, respectively. In a certain experiment, 8-nitroG and 8-oxodG were detected by double immunofluorescent technique. Then the cells were incubated with Alexa 594-labeled goat anti-rabbit IgG antibody and, when necessary, Alexa 488-labeled goat anti-mouse IgG antibody (1:400, Molecular Probes, Eugene, OR, USA) for 3 h. The nuclei were stained with 5 μM Hoechst 33258 (Polysciences Inc., Warrington, PA, USA). The stained cells were examined under a florescent microscope (BX53, Olympus, Tokyo, Japan). The staining intensity of 8-nitroG or iNOS per cell was quantified by analyzing 3-5 randomly selected fields per sample with an ImageJ software.

To examine the colocalization of HMGB1, RAGE, TLR9 and lysosome-associated membrane protein (LAMP) 1, we performed double immunoflurorescent techniques. A549 cells were treated with 1 μg/mL MWCNT for 8 h, and then incubated with mouse monoclonal antibody [anti-HMGB1 or anti-RAGE antibody (Abcam, 10 μg/ml)] and rabbit polyclonal antibody [anti-TLR9 (Santa Cruz Biotechnology, Santa Cruz, CA, USA, sc-25468, 2 μg/ml), anti-LAMP1 (Abcam, ab24170, 1 μg/ml) or anti-HMGB1 (Abcam, ab18256, 1 μg/ml) antibody] overnight at room temperature. The cells were incubated with Alexa 488-labeled goat anti-mouse IgG antibody and Alexa 594-labeled goat anti-rabbit IgG antibody and then examined as described above.

### Analysis of 8-nitroG formation induced by culture supernatant of MWCNT-exposed cells

We examined DNA-damaging ability of the molecules released from MWCNT-exposed cells as follows. A549 cells (5 × 10^5^ cells/ml) were treated with no or 1 μg/ml of MWCNT for 8 h at 37 °C in DMEM containing 100 mg/l kanamycin. Then we centrifuged the culture supernatant at 40,000 × *g* for 10 min at 4 °C to remove MWCNT. The supernatant was given to fresh A549 cells, followed by the incubation for 2 h at 37 °C. We also prepared the cells incubated in fresh DMEM as a negative control. 8-NitroG formation was examined by fluorescent immunocytochemistry as described above.

### Analysis of NO released from MNCNT-treated cells

To analyze NO release from MWCNT-treated cells, we measured the concentration of its products, nitrite (NO_2_^-^) plus nitrate (NO_3_^-^), in the culture supernatant using the Griess method. A549 cells (5 × 10^5^ cells/ml) were treated with 1 μg/ml of MWCNT for indicated durations at 37 °C in phenol red-free DMEM (Gibco) containing 5 % (v/v) FBS and 100 mg/l kanamycin. Then the culture supernatant was centrifuged at 40,000 × *g* for 10 min at 4 °C to remove MWCNT. To reduce NO_3_^-^ to NO_2_^-^, the supernatant was incubated with 0.1 units/ml of nitrate reductase from *Aspergillus niger* (Sigma-Aldrich) in the presence of 1 mM glucose-6-phosphate (Wako Pure Chemical Industries, Osaka, Japan), 0.3 units/ml of glucose-6-phosphate dehydrogenase and 20 μM NADPH (Oriental Yeast, Tokyo, Japan) for 30 min at room temperature. The reaction mixture was incubated with 0.25 % (w/v) sulfanilamide (Griess reagent I, Wako) and 0.025 % (w/v) naphthylethylenediamine (Griess reagent II, Sigma-Aldrich) in 0.625 % (v/v) phosphoric acid for 10 min at room temperature. The absorbance at 540 nm was measured with a Model 680 microplate reader (Bio-Rad Laboratories), and NO_2_^-^ concentration was determined by comparison with a standard curve generated with sodium nitrite (NaNO_2_, Wako).

### Measurement of GSH contents in MWCNT-exposed cells

Glutathione (GSH) contents in MWCNT-treated cells were measured by our method with slight modification [37]. A549 cells (5 × 10^5^ cells/ml) were treated with 1 μg/ml of MWCNT for indicated durations at 37 °C in DMEM containing 5 % (v/v) FBS and 100 mg/l kanamycin. The cells were lysed in cell lysis buffer (Cell Signaling Technology, Danvers, MA, USA) and sonicated briefly. The lysate was centrifuged at 14,000 × *g* for 10 min at 4 °C, and protein concentration in the supernatant was measured with a Coomassie Protein Assay Reagent Kit (Pierce Biotechnology, Rockford, USA). To precipitate proteins, 5 % (w/v) trichloroacetic acid was added to the same volume of the cell extract, and centrifuged at 18,000 × *g* for 10 min at 4 °C. The supernatant was diluted with 0.1 N HCl and analyzed with high-performance liquid chromatography (HPLC) coupled with an electrochemical detector (ECD, ECD-300, Eicom, Kyoto, Japan). GSH content was normalized with protein content.

### Measurement of ROS generation by flow cytometry

We measured peroxide levels in MWCNT-treated cells by flow cytometry as reported previously [38]. A549 cells (5 × 10^5^ cells/ml) were treated with 1 μg/ml of MWCNT for indicated durations at 37 °C in DMEM containing 5 % (v/v) FBS and 100 mg/l kanamycin. Five μM 5-(and-6)-chloromethyl-2’,7’-dichlorodihydrofluorescein diacetate acetyl ester (CM-H_2_DCFDA, Molecular Probes) was added 30 min before the end of the incubation to measure intracellular peroxide levels. The cells were suspended in PBS and analyzed with a FACScan flow cytometer (Becton Dickinson, San Jose, CA, USA).

### Measurement of 8-oxodG amount in MNCNT-treated cells

The levels of 8-oxodG in MNCNT-treated cells were measured by previously described method with modification [39]. A549 cells (5 × 10^5^ cells/ml) were treated with 1 μg/ml of MWCNT for indicated durations at 37 °C in DMEM containing 5 % (v/v) FBS and 100 mg/l kanamycin. The cells were lysed and treated with Proteinase K (Roche, Mannheim, Germany) for 1 h at 37 °C, and then sodium iodide was added. DNA was extracted and digested with nuclease P1 (Wako) and bacterial alkaline phosphatase (Sigma-Aldrich) to deoxynucleosides, and analyzed using HPLC coupled with an ECD (Coulochem II 5200A, ESA Biosciences, Chelmsford, MA, USA). The molar ratio of 8-oxodG to 2’-deoxyguanosine was measured based on ECD peak height of authentic 8-oxodG and the UV absorbance of 2’-deoxyguanosine at 254 nm.

### Electron microscopy

We investigated the intracellular distribution of MWCNT by transmission electron microscopy (TEM) as described previously [25]. A549 cells (5 × 10^5^ cells/ml) were treated with 1 μg/ml of MWCNT for 4 h at 37 °C in DMEM containing 5 % (v/v) FBS and 100 mg/l kanamycin. In certain experiments, the cells were pretreated with 2 mM MBCD or 50 μM MDC for 30 min. Cells were fixed with 2 % paraformaldehyde and 2 % glutaraldehyde in 0.1 M phosphate buffer (pH 7.4) for 90 min, and then treated with 1 % osmium tetroxide in 0.1 M phosphate buffer (pH 7.4) for 1 h. The cells were dehydrated in ethanol and acetone, and embedded in epoxy resin. Ultrathin sections (70 nm) were counterstained in 4 % uranyl acetate and lead acetate, and examined with a transmission electron microscope H-7000 (Hitachi, Tokyo, Japan).

### Transfection of cultured cells with siRNA

To investigate the role of TLR9 in MWCNT-induced DNA damage, A549 cells were transfected with 10 nM TLR9 siRNA (s28872 or s28873, Silencer® Select, Ambion) by using Lipofectamine® RNAiMAX reagent (Invitrogen) in Opti-MEM® I medium (Gibco), and incubated for 2 days at 37 °C. To exclude the possibility of off-target effects, cells were transfected with 10 nM Negative Control #2 siRNA (Ambion) instead. Depletion of TLR9 expression by siRNA was confirmed by fluorescent immunocytochemistry as described above and Western blotting as described below. In Western blotting, we used anti-TLR9 (1 μg/ml, Santa Cruz Biotechnology, sc-25468) and anti-GAPDH (0.2 μg/ml, Santa Cruz Biotechnology, sc-25778) rabbit polyclonal antibodies as primary antibodies and horseradish peroxidase (HRP)-conjugated donkey anti-rabbit IgG antibody (0.2 μg/ml, Santa Cruz Biotechnology) as the secondary antibody. Quantitative image analysis was performed with an ImageJ software.

### Quantitation of HMGB1 and DNA in culture supernatant of MWCNT-treated cells

We measured the amount of HMGB1 and double-stranded DNA (dsDNA) released from MWCNT-treated cells into the culture supernatant. A549 cells (5 × 10^5^ cells/ml) were treated with 1 μg/ml of MWCNT for indicated durations at 37 °C in DMEM containing 100 mg/l kanamycin. The culture supernatant was centrifuged at 40,000 × *g* for 10 min at 4 °C to remove MWCNT, and the supernatant was used for analysis. HMGB1 concentration was quantified with an HMGB1 ELISA kit II (Shino-test Corporation, Sagamihara, Japan) according to manufacturer’s instruction. To measure the amount of dsDNA, the supernatant was mixed with a QuantFluor dsDNA Dye (Promega Corporation, Madison, WI, USA) and analyzed with a Quantus Fluorometer (Promega Corporation).

### Co-immunoprecipitation and Western blotting

To confirm the association of TLR9 with HMGB1 and RAGE in MWCNT-exposed cells, we performed co-immunoprecipitation and Western blotting. A549 cells (5 × 10^5^ cells/ml) were treated with 1 μg/ml of MWCNT for 8 h at 37 °C in DMEM containing 5 % (v/v) FBS and 100 mg/l kanamycin. The cells were lysed in cell lysis buffer (Cell Signaling Technology) and sonicated briefly. The lysate was centrifuged at 14,000 × *g* for 10 min at 4 °C, and the supernatant was used for the experiment. Protein concentration was measured as described above. The cell lysate containing 400 μg of proteins was incubated with 2 μg of anti-TLR9 rabbit polyclonal antibody (Santa Cruz Biotechnology) overnight at 4 °C, followed by incubation with protein A magnetic beads (Cell Signaling Technology) for 20 min at room temperature. The samples were boiled for 5 min, separated by 5-20 % SDS-PAGE and blotted onto a polyvinylidene difluoride membrane. The membrane was treated with 5 % (w/v) skim milk in Tris-buffered saline (pH 7.4) containing 0.1 % (v/v) Tween 20, followed by the incubation with anti-TLR9 (0.5 μg/ml, Santa Cruz Biotechnology, sc-47723), anti-RAGE (2 μg/ml, Abcam) and anti-HMGB1 (2 μg/ml, Abcam) mouse monoclonal antibodies for 60 min and then with HRP-conjugated goat anti-mouse IgG antibody (1:2000, Santa Cruz Biotechnology) for 30 min. The membrane was incubated with ECL plus Western blotting detection reagents (GE Healthcare, Backinghamshire, UK) and analyzed with a LAS-4000 mini biomolecular imager (Fujifilm, Tokyo, Japan).

### Statistical analysis

Statistical analysis was performed by one-way or two-way analysis of variance (ANOVA) followed by Tukey’s multiple comparison test using an SPSS software (20.0 for Mac). Results were presented as means ± SD. *p* values less than 0.05 were considered to be statistically significant.

## Results

### Characteristics and size distribution of MWCNT agglomerates

**Fig. 1:**
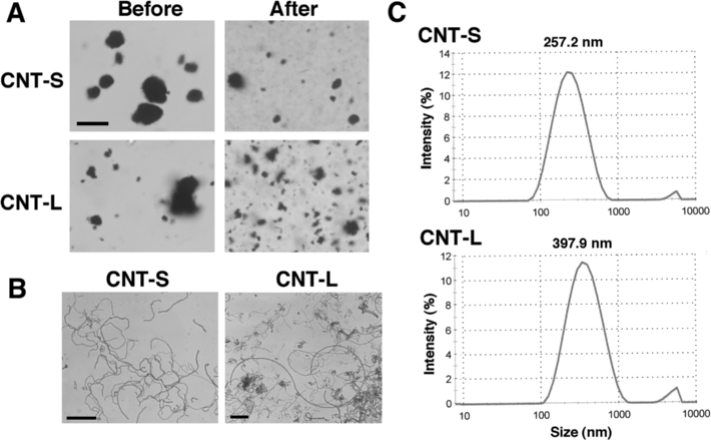
Dispersion of MWCNT agglomerates. MWCNT was suspended in DMEM and sonicated as described in Methods. **a** Photographs of MWCNT agglomerates before and after the sonication. The particles were observed with a light microscope. Bar = 10 μm. **b** Photographs of MWCNT fibers. After the sonication, MWCNT was observed with TEM as described in Methods. Bars = 1 μm. **c** Size distribution of dispersed MWCNT agglomerates. The size distribution was measured with a Zetasizer Nano particle size analyzer (Malvern, Worcestershire, UK)

In this study, we used two types of MWCNTs with different lengths [1-2 μm (CNT-S) and 5-15 μm (CNT-L)] and diameter of 20-40 nm. Trace elements contained in MWCNTs were analyzed with ICP-AES and ICP-MS. Detected trace metals are listed in Table 1 (full metal analysis is shown in Additional file 1: Table S1). We dispersed MWCNTs by sonication in PBS, dispersion medium [13] and DMEM containing 5 % (v/v) FBS. MNCNT agglomerates were most efficiently dispersed in DMEM among these media (Additional file 2: Figure S1), and they were used for further experiments. Figure 1 shows the photographs of MWCNT agglomerates obtained by sonication of their suspensions in DMEM. Light microscopy revealed that most MWCNT agglomerates were dispersed into submicron-sized particles (Fig. 1a). Figure 1b shows MWCNT fibers observed with TEM. The size distribution of MWCNT agglomerates is shown in Fig. 1c. The mean diameters of CNT-S and CNT-L agglomerates were 257.2 nm and 397.9 nm, respectively.

**Table 1:** Trace metals detected in MWCNTs used in this study (ND: not detected)

Element	CNT-S (ppm)	CNT-L (ppm)	Detection limit (ppm)
Na	ND	40	20
Mg	ND	300	10
Al	ND	30	10
Ca	30	100	20
Cr	ND	400	10
Mn	ND	30	10
Fe	50	2,000	10
Co	30	10	10
Ni	4,000	2,000	10
Mo	50	15,000	10
La	20	10	10

### Cytotoxic effect of MWCNT

**Fig. 2:**
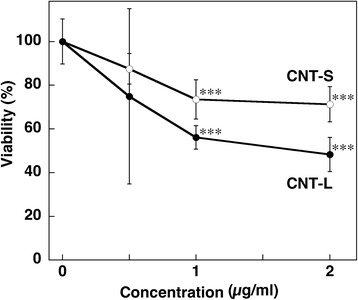
Cytotoxic effect of MWCNT. A549 cells were treated with 1 μg/ml MWCNT for 24 h at 37 °C, and the cell viability was examined by MTT assay as described in Methods. Viability of the control cells was set at 100 %. Data represent means ± SD of 4-6 independent experiments. ***p < 0.001, compared with the control by two-way ANOVA followed by Tukey’s test

A cytotoxic effect of MWCNT on A549 human lung epithelial cells was evaluated by MTT assay. Both CNT-S and CNT-L decreased the cell viability in a dose-dependent manner, and CNT-L tended to show a stronger cytotoxic effect than CNT-S (Fig. 2). Two-way ANOVA revealed that MWCNT concentrations (*F* = 12.093, *p* < 0.001) and length (CNT-S and CNT-L) (*F* = 5.551, *p* = 0.026) significantly influenced the cell viability, and there was no significant interaction between these factors (*F* = 0.901, *p* = 0.454). Tukey’s test revealed that MWCNTs significantly reduced the cell viability at 1 and 2 μg/ml compared with the control (*p* < 0.001, Fig. 2). UV-visible spectroscopy revealed that 1 μg/ml MWCNT had very low absorbance (Additional file 3: Figure S2A), and that the addition of MWCNT did not affect the spectrum of MTT (Additional file 3: Figure S2B). Therefore, the absorption and adsorption effects of MWCNTs in this assay appear to be negligible.

### DNA damage and iNOS expression in MWCNT-treated cells

**Fig. 3:**
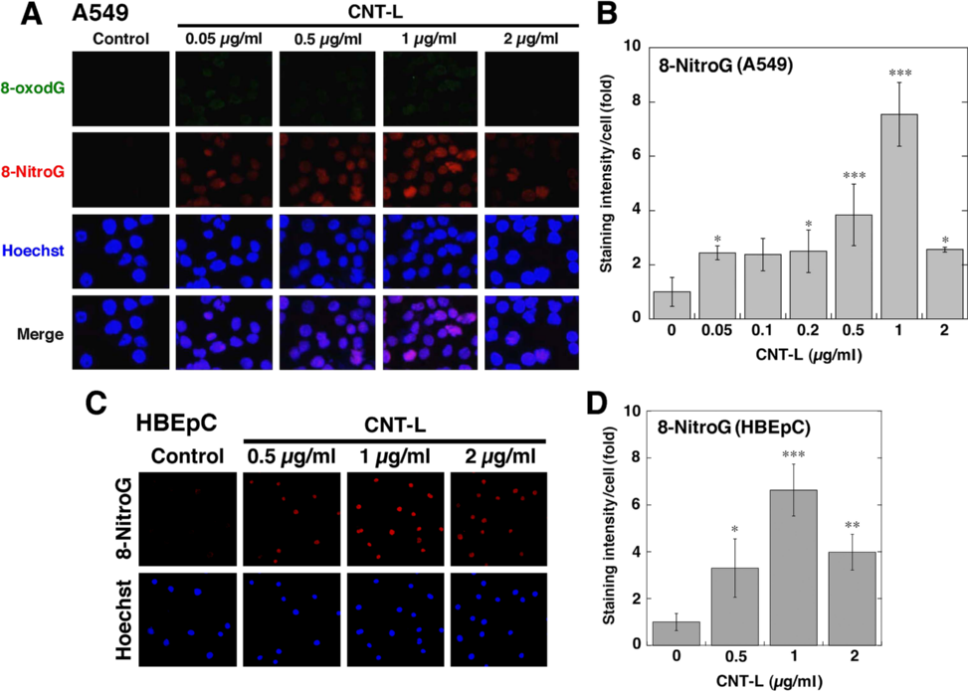
DNA damage in CNT-L-treated cells. **a** Immunofluorescent images of CNT-L-induced 8-oxodG and 8-nitroG formation in A549 cells. A549 cells were treated with CNT-L at indicated concentrations for 8 h at 37 °C, and 8-oxodG and 8-nitroG formation was examined by double immunofluorescent technique as described in Methods. Hoechst, Hoechst 33258. Magnification, X200. **b** Relative staining intensity of 8-nitroG formed in CNT-L-treated A549 cells. Staining intensity per cell was analyzed by an ImageJ software. Relative staining intensity of the control was set at 1. Data represent means ± SD of 8 (control) or 4 (CNT-L) independent experiments. *p < 0.05 and ***p < 0.001, compared with the control. **c** 8-NitroG formation in CNT-L-treated HBEpC cells. HBEpC cells were treated with CNT-L at indicated concentrations for 4 h at 37 °C, and immunofluorescent technique was performed. Magnification, X100. **d** Relative staining intensity of 8-nitroG formed in CNT-L-treated HBEpC cells. Data were quantitatively analyzed as described in (**b**). Data represent means ± SD of 3-4 independent experiments. *p < 0.05, **p < 0.01 and ***p < 0.001, compared with the control. Statistical analysis was performed by ANOVA followed by Tukey’s test

To investigate DNA damage in MWCNT-treated A549 cells, we performed double immunofluorescent technique, and the results are shown in Fig. 3. Figure 3a shows the formation of 8-oxodG and 8-nitroG in CNT-L-treated cells. Although no or weak staining of 8-oxodG was observed under the conditions used, the immunoreactivity of 8-nitroG was increased depending on MWCNT concentrations up to 1 μg/ml. No or weak staining was observed in non-treated control (Fig. 3a). 8-NitroG was formed mainly in the nucleus, which was stained with Hoechst 33258. Quantitative image analysis revealed that the staining intensity of 8-nitroG per cell was significantly greater in CNT-L-treated cells at 0.05-2 μg/ml (14-570 ng/cm^2^) than in non-treated control (*p* < 0.05 by ANOVA + Tukey’s test) and highest at 1 μg/ml (Fig. 3b). CNT-S induced 8-nitroG formation in a dose-dependent manner with statistical significance at 1 and 2 μg/ml (*p* < 0.001, Additional file 4: Figures S3A and S3B). MWCNTs also induced 8-nitroG formation in the nucleus of HBEpC, a normal human bronchial epithelial cell line (Fig. 3c and Additional file 4: Figure S3C). Image analysis revealed that MWCNTs significantly increased 8-nitroG formation in HBEpC cells (*p* < 0.05) and showed a similar dose-response relationship to A549 cells (Fig. 3d and Additional file 4: Figure S3D).

**Fig. 4:**
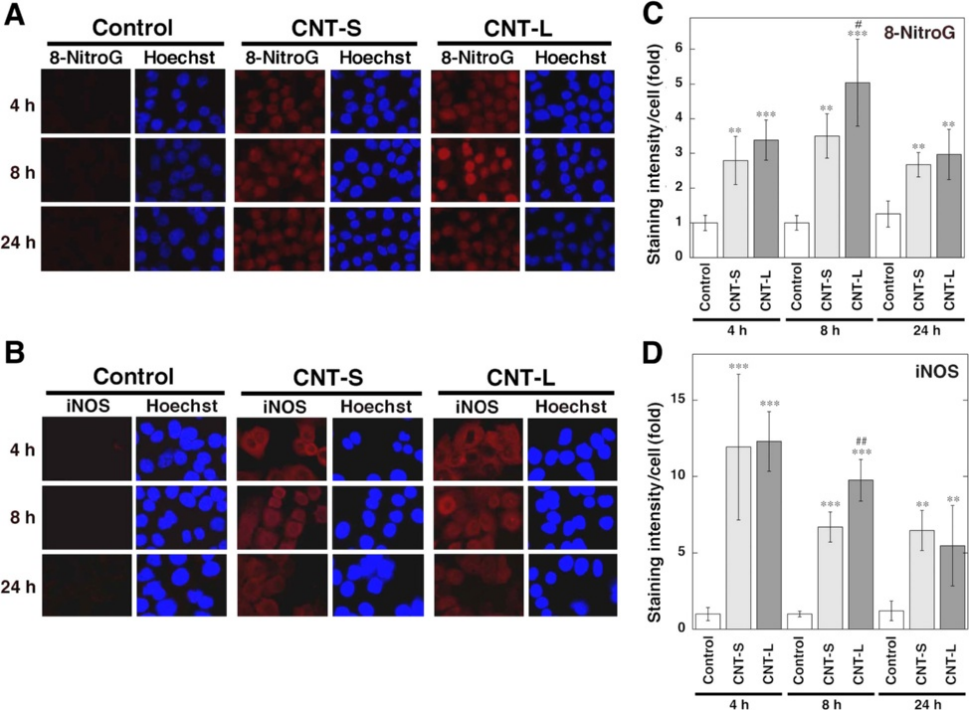
Time course of 8-nitroG formation and iNOS expression in MWCNT-treated cells. Immunofluorescent images of 8-nitroG formation (**a**) and iNOS expression (**b**) in MWCNT-treated A549 cells. A549 cells were treated with 1 μg/ml MWCNT for indicated durations at 37 °C, and 8-nitroG formation and iNOS expression were examined by fluorescent immunocytochemistry as described in Methods. Hoechst, Hoechst 33258. Magnification, X200. (**c**, **d**) Relative staining intensity of 8-nitroG and iNOS in A549 cells. Staining intensities of 8-nitroG (**c**) and iNOS (**d**) per cell were analyzed by an ImageJ software. Relative staining intensity of the control at 4 h was set at 1. Data represent means ± SD of 4 (control) and 6 (CNT-S and CNT-L) independent experiments. **p < 0.01 and ***p < 0.001, compared with the control; ^#^ *p* < 0.05 and ^##^ *p* < 0.01, compared with CNT-S-treated cells by ANOVA followed by Tukey’s test

We examined the time course of MWCNT-induced 8-nitroG formation and iNOS expression in A549 cells. Both CNT-S and CNT-L (1 μg/ml) increased 8-nitroG formation and iNOS expression at 4-24 h (Fig. 4a and b). In quantitative image analysis, the staining intensities of these molecules were significantly higher than those of the control throughout the experiments (*p* < 0.01, Fig. 4c and d). CNT-L induced significantly stronger 8-nitroG formation (*p* < 0.05) and iNOS expression (*p* < 0.01) than CNT-S at 8 h (Fig. 4c and d).

We also examined 8-nitroG formation in A549 cells treated with trace metals contained in MWCNTs at relatively high levels. Iron (FeCl_3_), nickel (NiCl_2_) and molybdenum compounds (MoCl_5_) and their combination did not induce 8-nitroG formation at the doses equivalent to those contained in 1 μg/ml MWCNT (Additional file 5: Figure S4).

### NO production and GSH decrease in MWCNT-treated cells

**Fig. 5:**
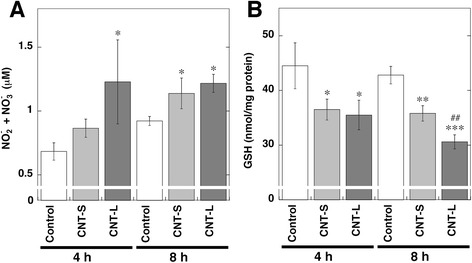
NO release and GSH decrease in MWCNT-treated cells. A549 cells were treated with 1 μg/ml MWCNT for indicated durations at 37 °C. **a** NO release from MWCNT-treated cells. The concentrations of NO_2_^-^ and NO_3_^-^ in culture supernatant were measured by the Griess method as described in Methods. **b** Decrease of GSH contents in MWCNT-treated cells. Intracellular GSH contents were measured with HPLC coupled with an ECD as described in Methods. Data represent means ± SD of 3 independent experiments. *p < 0.05, **p < 0.01 and ***p < 0.001, compared with the control; ^##^ *p* < 0.01, compared with CNT-S-treated cells by ANOVA followed by Tukey’s test

We measured the levels of NO products, NO_2_^-^ and NO_3_^-^, in culture supernatants of MWCNT-treated A549 cells using nitrate reductase and Griess reagents as shown in Fig. 5a. After the incubation for 4 h, CNT-L significantly increased NO release from the cells compared with the control (*p* < 0.01 by ANOVA + Tukey’s test). At 8 h, both CNT-S and CNT-L significantly increased NO release (*p* < 0.05). CNT-L tended to produce larger amount of NO compared with CNT-S. NO is known to readily reacts with GSH [40], and thus we examined the decrease of GSH contents in MWCNT-treated A549 cells using HPLC coupled with an ECD according to our previous study [37]. Both MWCNTs significantly reduced GSH contents at 4 and 8 h (*p* < 0.05), and CNT-L more greatly decreased GSH content than CNT-S at 8 h (*p* < 0.01, Fig. 5b).

### ROS generation and 8-oxodG formation in MWCNT-treated cells

To examine ROS generation in MWCNT-treated cells, we measured peroxide levels by flow cytometry using a fluorescent probe. Both CNT-S and CNT-L did not increase the fluorescent intensity at 4-24 h compared with the control (Additional file 6: Figure S5A). We quantified the amount of 8-oxodG using HPLC coupled with an ECD. CNT-S and CNT-L did not increase 8-oxodG formation at 4 and 8 h compared with the control (Additional file 6: Figure S5B).

### Intracellular distribution of MWCNT and effect of endocytosis inhibitors on 8-nitroG formation

**Fig. 6:**
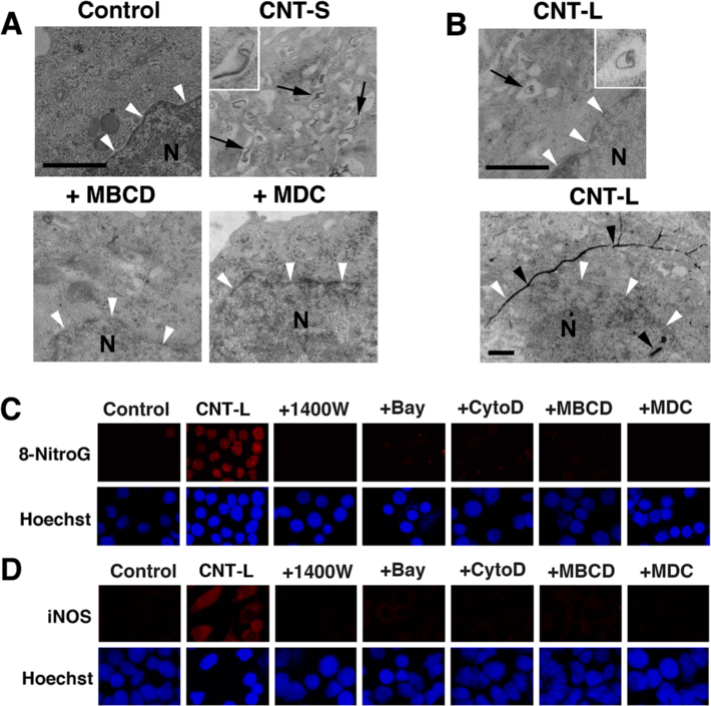
Intracellular distribution of MWCNT and effects of endocytosis inhibitors on inflammatory responses. **a** Intracellular distribution of CNT-S and effects of endocytosis inhibitors. A549 cells were incubated with 1 μg/ml of CNT-S for 4 h at 37 °C. In certain experiments, the cells were pretreated with 2 mM MBCD or 50 μM MDC for 30 min. Then the cells were observed by TEM as described in Methods. Numerous fibers can be seen in vesicular structures in the cytosol (arrows and inset), but no or few fibers are observed in MBCD- and MDC-pretreated cells. **b** Intracellular distribution of CNT-L. A549 cells were incubated with 1 μg/ml of CNT-L for 4 h at 37 °C. Only a few fibers are observed in vesicular structures in the cytosol (arrow and inset), and long fibers penetrated the plasma and nuclear membranes (black arrowheads). **a**, **b** Bars = 1 μm. N = nucleus. Nuclear membrane is indicated by white arrowheads. Effects of various inhibitors on CNT-L-induced 8-nitroG formation (**c**) and iNOS expression (**d**). A549 cells were treated with 1 μg/ml of CNT-L for 8 h at 37 °C in the presence of an inhibitor (1 μM 1400 W, 10 μM Bay, 1 μM CytoD, 2 mM MBCD or 50 μM MDC). Then, fluorescent immunocytochemistry was performed as described in Methods. Hoechst, Hoechst 33258. Magnification, X200

We examined the intracellular distribution of MWCNT in A549 cells by TEM as shown in Fig. 6a and b. Numerous short fibers were observed in vesicular structures in the cytosol of CNT-S-treated cells, and the pretreatment with MBCD, an inhibitor of caveolae-mediated endocytosis, and MDC, an inhibitor of clathrin-mediated endocytosis, largely reduced their internalization (Fig. 6a). On the other hand, a few short fibers were found in vesicles in the cytosol, and long fibers penetrated the plasma and nuclear membranes of CNT-L-treated cells (Fig. 6b).

We examined the effects of various inhibitors on MWCNT-induced 8-nitroG formation and iNOS expression by immunocytochemistry. CNT-L induced clear 8-nitroG formation (Fig. 6c) and iNOS expression (Fig. 6d), and their staining was almost completely attenuated by the treatment with 1400 W, an iNOS inhibitor, and Bay, an inhibitor of nuclear factor-κB (NF-κB) activation. The immunoreactivities of 8-nitroG and iNOS were also reduced by endocytosis inhibitors, MBCD, MDC and CytoD, an inhibitor of actin polymerization (Fig. 6c and d). Similar findings were observed with CNT-S-treated A549 cells (Additional file 7: Figure S6). These inhibitors did not show significant cytotoxicity in A549 cells under the conditions used (Additional file 8: Figure S7). Our previous study has demonstrated that these inhibitors effectively suppressed MWCNT-induced 8-nitroG formation and iNOS expression, and did not cause these molecular events by themselves [25].

### Involvement of TLR9 in MWCNT-induced 8-nitroG formation

**Fig. 7:**
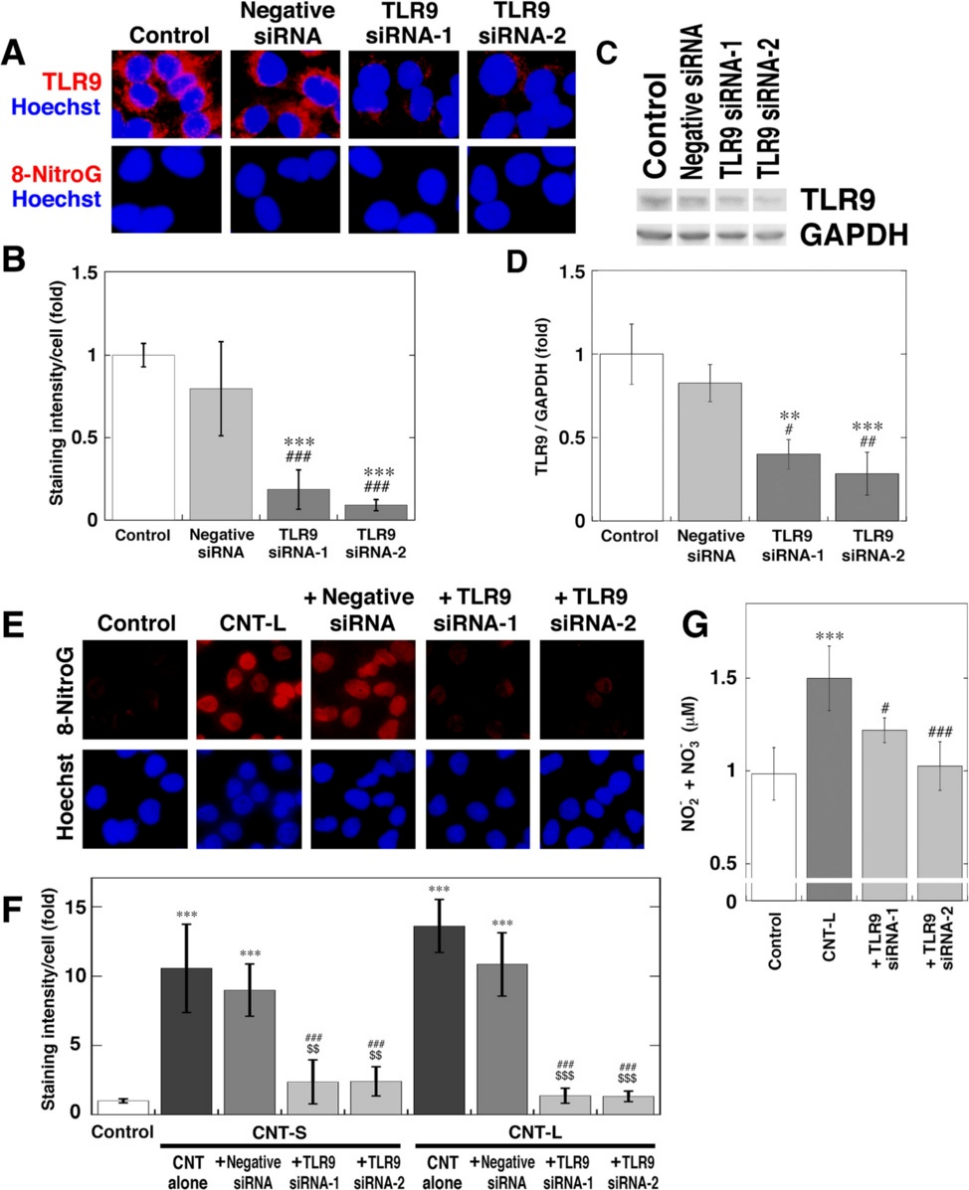
Inhibitory effect of TLR9 siRNA on MWCNT-induced 8-nitroG formation. **a** Immunofluorescent images of reduced TLR9 expression and the absence of 8-nitroG formation in siRNA-transfected cells. A549 cells were transfected with negative control or TLR9 siRNA (siRNA-1 and siRNA-2). TLR9 expression and 8-nitroG formation were examined by fluorescent immunocytochemistry. Hoechst, Hoechst 33258. Magnification, X400. **b** Relative staining intensity of TLR9 in siRNA-transfected A549 cells. The staining intensity of TLR9 per cell was analyzed by an ImageJ software. Relative staining intensity of the control was set at 1. Data represent means ± SD of 4 independent experiments. ***p < 0.001, compared with the control; ^###^ *p* < 0.001, compared with negative control siRNA-transfected cells. **c** Reduction of TLR9 expression in siRNA-transfected cells. A549 cells transfected with TLR9 siRNA were lysed and treated, and then Western blotting was performed to detect TLR9 and GAPDH as described in Methods. **d** Relative intensity of TLR9 expression in siRNA-transfected A549 cells. The relative band intensity of TLR9 normalized by GAPDH was measured with an ImageJ software. Data represent means ± SD of 3 independent experiments. **p < 0.01 and ***p < 0.001, compared with the control; ^#^ *p* < 0.05 and ^##^ *p* < 0.01, compared with negative control siRNA-transfected cells. **e** Inhibitory effect of TLR9 siRNA on MWCNT-induced 8-nitroG formation. After siRNA transfection, A549 cells were treated with 1 μg/ml CNT-L for 8 h. 8-NitroG formation was analyzed by fluorescent immunocytochemistry. Magnification, X200. **f** Quantitative image analysis for effects of TLR9 siRNA on MWCNT-induced 8-nitroG formation. Staining intensity was analyzed as described in (**b**). Data represent means ± SD of 3-4 independent experiments. ***p < 0.001, compared with the control, ^###^ *p* < 0.001, compared with MWCNT alone, ^$$^ *p* < 0.01 and ^$$$^ *p* < 0.001, compared with MWCNT + negative control siRNA. **g** Effect of TLR9 siRNA on NO release from MWCNT-exposed cells. After siRNA transfection, A549 cells were treated with 1 μg/ml CNT-L for 4 h. Data represent means ± SD of 5-6 independent experiments. ***p < 0.001, compared with the control; ^#^ *p* < 0.05 and ^###^ *p* < 0.001, compared with CNT-L-treated cells. Statistical analysis was performed by ANOVA followed by Tukey’s test

To clarify the role of TLR9 in MWCNT-induced 8-nitroG formation, we examined the inhibitory effect of siRNA transfection by immunocytochemistry. We used two types of TLR9 siRNA [s28872 (siRNA-1) and s28873 (siRNA-2)] that interact with different sites of mRNA. TLR9 expression was observed in the cytosol of non-treated A549 cells, and largely reduced by transfection with both types of siRNA (Fig. 7a). Negative control siRNA did not affect TLR9 expression (Fig. 7a). We confirmed that negative control or TLR9 siRNA did not induce 8-nitroG formation (Fig. 7a). Quantitative image analysis showed that the staining intensity of TLR9 was significantly lower in siRNA-transfected cells than in the control and negative control siRNA-transfected cells (*p* < 0.001, Fig. 7b). Western blotting also revealed that siRNAs significantly reduced TLR9 expression compared with the control and negative control siRNA-treated cells (Fig. 7c and d). TLR9 siRNAs largely attenuated CNT-L-induced 8-nitroG formation in A549 cells, but negative control siRNA did not (Fig. 7e). Similar inhibitory effect of TLR9 siRNA was observed with CNT-S-induced A549 cells (data not shown). Quantitative image analysis revealed that the transfection with both types of TLR9 siRNA significantly reduced the staining intensity of 8-nitroG formed by CNT-S and CNT-L (*p* < 0.001, Fig. 7f). TLR9 siRNA significantly decreased NO release from MWCNT-treated cells (*p* < 0.05, Fig. 7g). Transfection with TLR9 siRNA also reduced 8-nitroG formation in MWCNT-exposed HBEpC cells (Additional file 9: Figure S8). Negative control or TLR9 siRNA alone did not induce 8-nitroG formation in HBEpC cells (Additional file 9: Figure S8).

### Involvement of HMGB1 and RAGE in MWCNT-induced 8-nitroG formation

**Fig. 8:**
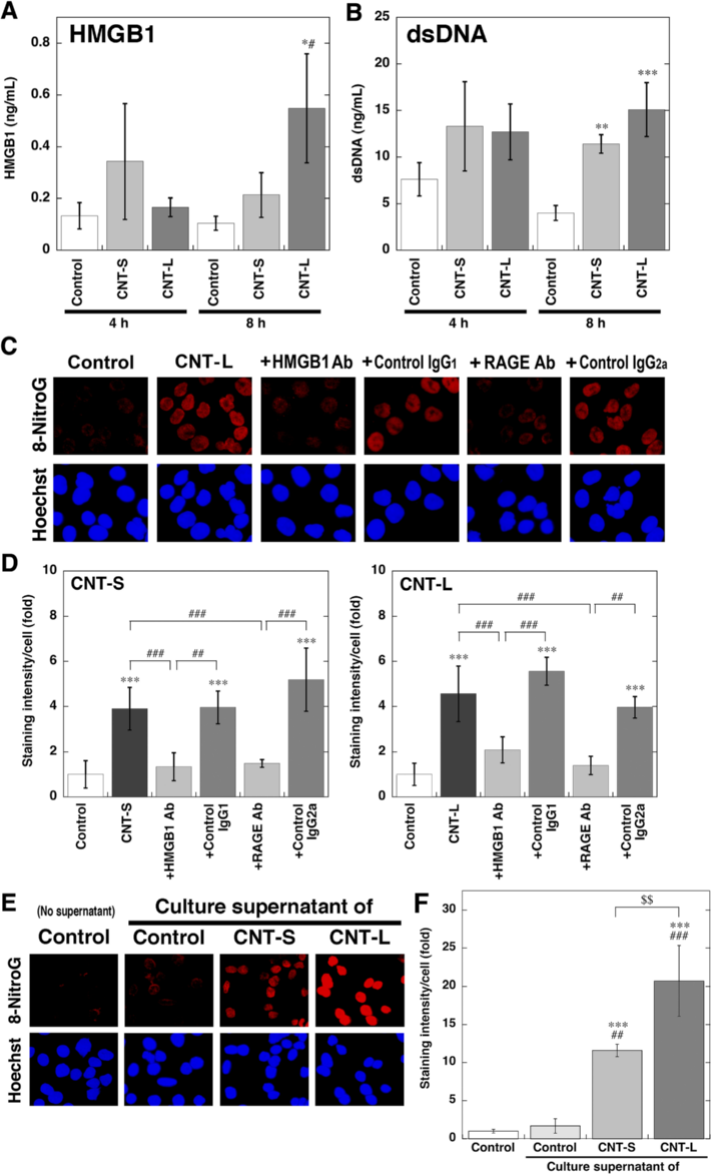
Release of HMGB1 and dsDNA from MWCNT-treated cells and inhibitory effects of antibodies on 8-nitroG formation. **a** Release of HMGB1 from MWCNT-treated cells. A549 cells were treated with 1 μg/ml of MWCNT for indicated durations at 37 °C. Then the concentration of HMGB1 in the culture supernatant was measured by a commercial ELISA kit. **b** Release of dsDNA from MWCNT-treated cells. A549 cells were treated with 1 μg/ml of MWCNT at 37 °C, and the concentration of dsDNA in the culture supernatant was measured with a Quantus fluorometer. **a**, **b** Data represent means ± SD of 3 independent experiments. *p < 0.05, **p < 0.01 and ***p < 0.001 compared with the control; ^#^ *p* < 0.05, compared with CNT-S-treated cells. **c** Inhibitory effects of anti-HMGB1 and anti-RAGE antibodies on MWCNT-induced 8-nitroG formation. A549 cells were pretreated with 10 μg/ml anti-HMGB1 or anti-RAGE antibody (or corresponding control IgG) for 30 min and then treated with 1 μg/ml CNT-L for 8 h. Then fluorescent immunocytochemistry was performed as described in Methods. Hoechst, Hoechst 33258. Magnification, X200. **d** Quantitative image analysis for effects of the antibodies on 8-nitroG formation in MWCNT-treated cells. Staining intensity per cell was analyzed by an ImageJ software. Relative staining intensity of the control was set at 1. Data represent means ± SD of 8 (control and MWCNT alone) or 4 (MWCNT plus an antibody) independent experiments. ***p < 0.001, compared with the control; ^##^ *p* < 0.01 and ^###^ *p* < 0.001. **e** 8-NitroG formation induced by culture supernatant of MWCNT-exposed cells. A549 cells were treated with 1 μg/ml of MWCNT for 8 h, and the culture supernatant was given to fresh A549 cells, followed by incubation for 2 h at 37 °C. 8-NitroG formation was examined by fluorescent immunocytochemistry. **f** Quantitative image analysis for 8-nitroG formation induced by culture supernatant of MWCNT-treated cells. Staining intensity was analyzed as described in (**d**). Data represent means ± SD of 3-4 independent experiments. ***p < 0.001, compared with the non-treated control; ^##^ *p* < 0.01 and ^###^ *p* < 0.001, compared with the cells treated with control cell supernatant; ^$$^ *p* < 0.01. Statistical analysis was performed by ANOVA followed by Tukey’s test

We examined the role of the HMGB1-RAGE interaction in MWCNT-induced 8-nitroG formation. We quantified the amounts of HMGB1 in the culture supernatant of MWCNT-treated A549 cells by ELISA. The treatment with CNT-S non-significantly increased HMGB1 concentration in the culture supernatant at 4 and 8 h (Fig. 8a). CNT-L significantly increased the release of HMGB1 from the cells at 8 h compared with the control and CNT-S (*p* < 0.05, Fig. 8a). We also measured the concentration of dsDNA in the culture supernatant with fluorometric analysis. Both MWCNTs nonsignificantly and significantly (*p* < 0.01) increased the release of dsDNA from the cells at 4 and 8 h, respectively (Fig. 8b). Fluorescent immunohistochemistry revealed that the pretreatment of the cells with anti-HMGB1 and anti-RAGE antibodies reduced CNT-L-induced 8-nitroG formation (Fig. 8c). The addition of their isotype control IgGs (IgG_1_ and IgG_2a_ for anti-HMGB1 and anti-RAGE antibodies, respectively) did not affect 8-nitroG formation (Fig. 8c). Anti-HMGB1 and anti-RAGE antibodies showed similar inhibitory effects on CNT-S-induced 8-nitroG formation (data not shown). Quantitative image analysis revealed that the addition of anti-HMGB1 and anti-RAGE antibodies significantly reduced the staining intensity of 8-nitroG in CNT-S- and CNT-L-treated cells (*p* < 0.001, Fig. 8d). We confirmed that these antibodies and control IgGs did not show significant cytotoxicity in A549 cells under the conditions used (Additional file 10: Figure S9). These antibodies also reduced 8-nitroG formation in MWCNT-exposed HBEpC cells (Additional file 11: Figure S10).

We examined whether the culture supernatant of MWCNT-exposed A549 cells induces 8-nitroG formation in fresh cells. No or very weak 8-nitroG formation was observed in control cells not treated with culture supernatant. The supernatant of MWCNT-exposed cells clearly induced 8-nitroG formation, whereas the supernatant of non-treated cells induced only slight 8-nitroG formation (Fig. 8e). Image analysis revealed that the supernatant of MWCNT-exposed cells significantly increased its staining intensity compared with the control (*p* < 0.001) and the cells treated with control cell supernatant (*p* < 0.01, Fig. 8 f). The supernatant of CNT-L-exposed cells induced significantly stronger 8-nitroG formation than that of CNT-S-exposed cells (*p* < 0.01, Fig. 8 f).

HMGB1 has been reported to interact with TLR2 and TLR4 on the cell surface to induce inflammatory responses [33]. We examined the effects of anti-TLR2 and anti-TLR4 antibodies on MWCNT-induced 8-nitroG formation, but these antibodies did not reduce 8-nitroG formation (Additional file 12: Figure S11).

### Association of HMGB1, RAGE and TLR9 in MWCNT-treated cells

**Fig. 9:**
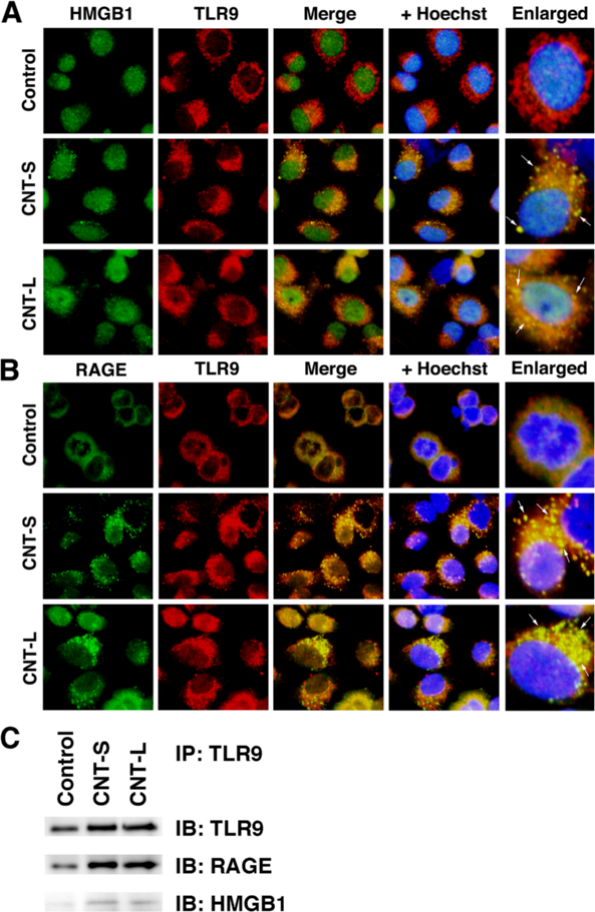
Association of TLR9 with HMGB1 and RAGE in MWCNT-treated cells. A549 cells were treated with 1 μg/mL MWCNT for 8 h. Double immunofluorescent analysis was performed to examine the colocalization of TLR9 with HMGB1 (**a**) and RAGE (**b**) as described in Methods. HMGB1 and RAGE were stained with the corresponding mouse monoclonal antibodies and Alexa 488-labeled goat anti-mouse IgG antibody. TLR9 was stained with the rabbit polyclonal antibody and Alexa 594-labeled goat anti-rabbit IgG antibody. Hoechst, Hoechst 33258. Magnification, X400. Arrows indicate the colocalization of TLR9 with HMGB1 or RAGE in the cytosol. **c** Interaction of TLR9 with RAGE and HMGB1. Immunoprecipitation (IP) was performed using anti-TLR9 rabbit polyclonal antibody and TLR9, RAGE and HMGB1 were detected by Western immunoblotting (IB). The membrane was treated with corresponding mouse monoclonal antibodies. Data are representative of 3 independent experiments

**Fig. 10:**
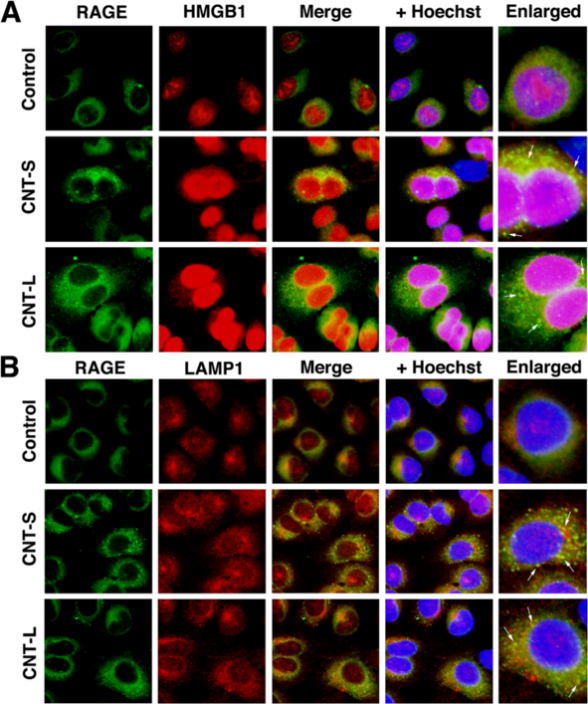
Colocalization of RAGE with HMGB1 and LAMP1 in MWCNT-treated cells. A549 cells were treated with 1 μg/mL MWCNT for 8 h, and then double immunofluorescent analysis was performed to examine the colocalization of RAGE with HMGB1 (**a**) and LAMP1, a lysosomal marker (**b**), as described in Methods. RAGE was stained with the mouse monoclonal antibody and Alexa 488-labeled goat anti-mouse IgG antibody. HMGB1 and LAMP were stained with the corresponding rabbit polyclonal antibodies and Alexa 594-labeled goat anti-rabbit IgG antibody. Hoechst, Hoechst 33258. Magnification, X400. Arrows indicate the colocalization of RAGE with HMGB1 or LAMP in the cytosol

Figures 9 and 10 show the localization of HMGB1, RAGE and TLR9 in MWCNT-treated cells examined by double immunofluorescent techniques. In non-treated control cells, HMGB1 was expressed in the nucleus, and did not colocalize with TLR9 in the cytosol (Fig. 9a). In MWCNT-treated cells, HMGB1 staining was also observed in the cytosol, and colocalized with TLR9 (Fig. 9a). RAGE staining was observed in the cell membrane and cytosol of control cells, but did not colocalize with TLR9 (Fig. 9b). In MWCNT-treated cells, RAGE exhibited a vesicular pattern in the cytosol and colocalized with TLR9 (Fig. 9b). Co-immnoprecipitation using anti-TLR9 antibody and Western blotting revealed that the band intensities of RAGE and HMGB1 were increased by MWCNT compared with the control, suggesting that TLR9 interacts with these molecules in MWCNT-exposed cells (Fig. 9c). The colocalization of RAGE and HMGB1 was observed in the cytosol of MWCNT-treated cells, but not in control cells (Fig. 10a). RAGE was colocalized with LAMP1, a lysosome marker, in MWCNT-treated cells (Fig. 10b). These results suggest that HMGB1 and RAGE undergo cellular internalization and interact with TLR9 in the lysosome of MWCNT-exposed cells.

## Discussion

We investigated MWCNT-induced DNA damage mediated by inflammatory responses in human lung epithelial cell lines, A549 and HBEpC, and its molecular mechanism. Inflammation is the primary source of RNS, and the formation of nitrative DNA lesions, including 8-nitroG, may contribute to MWCNT-induced carcinogenesis. We previously demonstrated that 8-nitroG was formed at the sites of inflammation-related carcinogenesis in animal models and clinical specimens [19–22]. The glycosidic bond between 8-nitroG and deoxyribose is chemically unstable and thus 8-nitroG can be spontaneously released to form an apurinic site [41]. During DNA synthesis, adenine is preferentially misincorporated opposite an apurinic site [42] and intact 8-nitroG [43], leading to G → T transversion [42]. Therefore, 8-nitroG is a potentially mutagenic DNA lesion.

In this study, immunocytochemistry and quantitative image analysis revealed that CNT-L significantly increased 8-nitroG formation at 0.05 μg/ml (equivalent to 14 ng/cm^2^) in A549 cells. According to a recent workplace exposure assessment [44] and a particle deposition model [45], we estimated that the level of CNT deposition in human alveoli reaches 14 ng/cm^2^ within 8 years (Table 2). Because this estimation was made by assuming that CNT is evenly distributed on the alveolar surface, CNT level may actually exceed this value at particular sites in a shorter duration. Therefore, our experimental conditions appear to be occupationally relevant. CNT-L induced maximal 8-nitroG formation at 1 μg/ml and its staining intensity was decreased at 2 μg/ml in both cell lines, probably due to a dose-dependent cytotoxic effect as demonstrated by MTT assay or fluorescence quenching by MWCNT that remained in cells. CNT-L temporarily caused significantly stronger 8-nitroG formation and iNOS expression in A549 cells than CNT-S at 8 h, although there was no significant difference thereafter. Previous studies demonstrated that long CNT fibers caused more severe histopathological changes and tumorigenicity than short fibers in experimental animals [5, 46]. These results suggest that inflammation-related DNA damage is partially involved in the effect of fiber length on CNT-induced carcinogenicity. CNT-S significantly increased 8-nitroG formation compared with the control, but the release of NO into the culture supernatant was not significantly increased at 4 h. This discrepancy is probably because a large part of intracellular NO reacts with biomolecules, including DNA bases and GSH, before the leakage into the extracellular space. MWCNT exposure significantly decreased intracellular GSH contents, and this finding supports NO production in cells.

**Table 2:** Estimation of alveolar MWCNT deposition in exposed individuals

Factors	Values	References
Mean of airborne MWCNT concentration	10.6 μg/m^3^	[44]
Minute ventilation	0.02 m^3^/min	
Diameter of MWCNT agglomerates	400 nm	Fig. 1c (in this study)
Alveolar deposition efficiency (dependent on diameter of agglomerates)	7 %	[45]
Alveolar surface area	100 m^2^	
Alveolar deposition of MWCNT		
10.6 μg/m^3^ × 0.02 m^3^/min × 7 %	=14.8 ng/min	
	=7.10 μg/day	(8 h/day)
	=35.5 μg/week	(5 days/week)
	=1.78 mg/year	(50 weeks/year)
	=1.78 ng/cm^2^/year	(per alveolar surface area)
MWCNT deposition reaches 14 ng/cm^2^ (at which 8-nitroG formation was increased) in		
(14 ng/cm^2^)/(1.78 ng/cm^2^/year)	=7.87 years	

MWCNT used in this study contained relatively high concentrations of iron, nickel and molybdenum, all of which are reported to be lung carcinogens [47–50]. In this study, we examined whether these trace metals induce DNA damage on the assumption that metal impurities were released from MWCNT into biological fluid. These metal compounds did not cause 8-nitroG formation, suggesting that metal impurities have no or weak ability to catalyze reactive species and cause DNA damage under the conditions used. It has been reported that iron and molybdenum were released from their particles in synthetic body fluid [51] and that nickel ions were released from nickel oxide nanoparticles in dispersion [52]. The bioavailability of metals used in this experiment appears to be comparable to those contained in MWCNT, although we cannot exclude the possibility that metal impurities remained insoluble in our experimental systems.

TEM revealed that short MWCNT fibers were found in vesicular structures in the cytosol. Their cellular internalization was suppressed by MBCD and MDC, inhibitors of caveolae- and clathrin-mediated endocytosis, respectively. Nanoparticles up to approximately 500 nm and 200 nm are primarily internalized by caveolae- and clathrin-mediated endocytosis, respectively [53, 54]. MWCNT used in this study may be internalized via endocytosis, because the mean diameters of their agglomerates were within 500 nm. Nanoparticles are considered to induce cell death via the cellular internalization and lysosomal dysfunction [55]. In addition, long MWCNT fibers were found in the cytosol and nucleus. There are observations that MWCNT pierced the plasma and nuclear membranes of lung epithelial [25] and mesothelial cells [7], in agreement with this study. However, there is a possibility that MWCNT was displaced during cutting the sections for TEM and appeared in the cytosol and nucleus, because MWCNT is a very hard material. In addition, MWCNT in lysosomes may leak out into the cytosol and interact with other organelles. Taken together, MWCNT appears to cause cell injury in endocytosis-dependent and possibly independent manners, although the precise mechanism remains to be clarified.

TLRs are responsible for inflammatory responses against a wide variety of infectious and noninfectious agents. TLR9 resides in endosomes and lysosomes, and activates inflammatory responses via interaction with CpG DNA of exogenous and endogenous origins [26]. In human lung tissues, TLR9 is expressed in alveolar macrophage, bronchial epithelium and alveolar septal cells [29]. HMGB1 is a nuclear protein, which is released from damaged or necrotic cells and associated with inflammatory diseases and cancer [32, 33]. The HMGB1-DNA complex binds to RAGE and then activates TLR9-mediated inflammatory responses [31]. In this study, the formation of the HMGB1-DNA complex is supported by the cytotoxicity of MWCNT and the release of HMGB1 and dsDNA from MWCNT-treated cells into the culture supernatant. This hypothesis is also supported by the finding that the culture supernatant of MWCNT-treated A549 cells significantly increased 8-nitroG formation in fresh cells. A previous study showed that non-treated A549 cells released DNA at similar levels to that measured in this study, and DNA release was increased with an increase in the number of dead cells [56]. MWCNT-induced 8-nitroG formation was attenuated by pretreatment with anti-HMGB1 and anti-RAGE antibodies and the transfection with TLR9 siRNA in both A549 and HBEpC cells, suggesting that the HMGB1-RAGE-TLR9 signaling pathway plays the key role in MWCNT-induced DNA damage. The involvement of TLR9 in DNA damage is supported by the inhibitory effect of siRNA on NO release from MWCNT-exposed cells. The addition of isotype control IgGs and the transfection with negative control siRNA did not affect 8-nitroG formation, confirming that the involvement of these molecules. Antibodies against TLR2 and TLR4, which are reported to interact with HMGB1 on cell surface [33], did not reduce MWCNT-induced 8-nitroG formation, suggesting that the contribution of these receptors to genotoxicity is small. MWCNT-induced 8-nitroG formation was attenuated by MBCD, MDC and CytoD, suggesting that the HMGB1-DNA complex is internalized into the cells via endocytosis. MBCD induces cholesterol depletion in the plasma membrane. Cholesterol accumulation promotes inflammatory responses, including activation of TLR signaling pathways [57]. MDC is used to stain autophagic vacuoles [58]. There is increasing evidence that autophagy participates in induction of inflammatory responses [59]. Therefore, the effects of these chemicals on MWCNT-induced DNA damage may be partially accounted for by endocytosis-independent mechanisms.

Double immunofluorescent analysis revealed that HMGB1, RAGE, TLR9 and the lysosomal marker LAMP1 were colocalized in the cytosol, indicating that these molecules interact in lysosomes. Co-immunoprecipitation and Western blotting confirmed that TLR9 was associated with HMGB1 and RAGE in MWCNT-exposed cells. TLR9 mediates NF-κB-dependent inflammatory responses [26] and NF-κB mediates the expression of iNOS and various cytokines [60, 61]. 8-NitroG formation was inhibited by 1400 W and Bay, indicating the involvement of NF-κB-dependent iNOS expression. Relevantly, asbestos induces inflammatory responses via an HMGB1-dependent pathway in cultured cells, experimental animals and exposed individuals [34]. HMGB1 and CpG oligonucleotides synergistically enhanced the proliferation and invasive potential of human lung cancer cells via the interaction with RAGE [62]. These findings support our hypothesis that TLR9-mediated signaling pathway contributes to inflammation and genotoxicity induced by MWCNT, although there remains a possibility that MWCNT induces HMGB1-mediated inflammatory responses via NLRP3 inflammasome as demonstrated by an in vivo experiment [63].

**Fig. 11:**
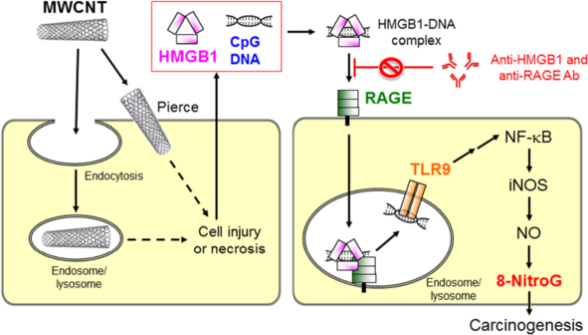
Proposed mechanism of MWCNT-induced DNA damage

On the basis of our results, the proposed mechanism of MWCNT-induced DNA damage is shown in Fig. 11. MWCNT causes cell injury or necrosis via endocytosis or penetration of the plasma membranes. HMGB1 and DNA are released from damaged cells and form the HMGB1-DNA complex, which interacts with RAGE to be internalized into neighboring living cells. CpG DNA bound to HMGB1 is recognized by TLR9 in the endosome and/or lysosomes. TLR9 mediates NO generation via NF-κB-dependent iNOS expression, resulting in nitrative DNA damage. This mechanism may contribute to MWCNT-induced carcinogenesis.

In this study, MWCNTs induced 8-nitroG formation and NO generation in cultured cells, but did not increase 8-oxodG formation and ROS generation. Physiological levels of O_2_^-^ and NO react to produce ONOO^-^ and its conjugate acid, ONOOH. ONOOH decomposes via homolysis to give •OH and •NO_2_, which cause oxidative and nitrative DNA damage, respectively. However, under physiological conditions, ONOO^-^ reacts with CO_2_ derived from bicarbonate to generate peroxynitrosocarbonate (ONOOCO_2_^-^), which undergoes homolysis to give •NO_2_ and •CO_3_^-^ [64, 65]. An in vitro experiment revealed that the addition of bicarbonate dose-dependently increased ONOO^-^-induced 8-nitroG formation (up to 10 mM), but had no effect on 8-oxoguanine formation, suggesting that CO_2_ potentiates 8-nitroG formation and inactivates •OH-like activity of ONOOH [66]. Although the cell culture medium used in this study (DMEM) contains sodium bicarbonate (NaHCO_3_) at a relatively high concentration (44 mM, disclosed by the manufacturer), bicarbonate abundantly exists under physiological conditions (approximately 25 mM) [67]. Therefore, MWCNTs appear to preferably induce 8-nitroG formation in biological systems.

## Conclusions

In this study, MWCNT induced nitrative DNA damage in human lung epithelial cell lines under the occupationally relevant conditions. The DNA damage involves the release of HMGB1 and DNA from damaged and/or necrotic cells due to MWCNT exposure. The interaction of the HMGB1-DNA complex with RAGE leads to TLR9 activation, which mediates NO generation and resulting DNA damage. This is the first study demonstrating that TLR9 and related molecules participate in MWCNT-induced genotoxicity. This study raises a possibility that 8-nitroG can be used as a biomarker to evaluate the carcinogenic risks of particulate and fibrous materials and that the HMGB1-RAGE-TLR9 pathway is a diagnostic and therapeutic target of diseases caused by these materials.

## Additional files

Additional file 1: Table S1. Full metal analysis for trace elements contained in MWCNTs used in this study. (DOC 77 kb)

Additional file 2: Figure S1. Dispersion of MWCNT agglomerates in different media. CNT-L was dispersed by sonication in PBS, dispersion medium (DM) and DMEM, and then observed with a light microscope as described in Methods. Agglomerates were much more efficiently dispersed in DMEM than in PBS and dispersion medium. Bar = 50 μm. (TIF 579 kb)

Additional file 3: Figure S2. Effect of MWCNT on UV-visible spectrum of MTT. (A) UV-visible spectra of MWCNT (CNT-S or CNT-L, 1 μg/ml). (B) Effect of MWCNT on the spectrum of MTT. The reaction mixture contained 0.05 mg/ml MTT, and 1 μg/ml CNT-S or CNT-L was added. The spectra were measured before (MTT) and immediately (0 min) and 20 min after the addition of MWCNT (+CNT-S and + CNT-L) at 25 °C. There was no spectral change at 0 and 20 min. (TIF 125 kb)

Additional file 4: Figure S3. DNA damage in CNT-S-treated cells. (A) Immunofluorescent images of 8-nitroG formation in CNT-S-treated A549 cells. A549 cells were incubated with CNT-S at indicated concentrations for 8 h at 37 °C, and 8-nitroG formation was examined by immunofluorescent technique as described in Methods. Hoechst, Hoechst 33258. Magnification, X200. (B) Relative staining intensity of 8-nitroG formed in CNT-S-treated A549 cells. Staining intensity per cell was analyzed by an ImageJ software. Relative staining intensity of the control was set at 1. Data represent means ± SD of 3 or 4 independent experiments. ***p < 0.001, compared with the control. (C) 8-NitroG formation in CNT-S-treated HBEpC cells. HBEpC cells were treated with CNT-S at indicated concentrations for 4 h at 37 °C, and immunofluorescent technique was performed. Magnification, X100. (D) Relative staining intensity of 8-nitroG formed in CNT-S-treated HBEpC cells. Staining intensity was analyzed as described in (B). Data represent means ± SD of 3 or 4 independent experiments. ***p < 0.001, compared with the control. Statistical analysis was performed by ANOVA followed by Tukey’s test. (TIF 654 kb)

Additional file 5: Figure S4. Effect of trace metals contained in MWCNT on 8-nitroG formation. A549 cells were treated with 2 ng/ml iron (36 nM FeCl_3_), 4 ng/ml nickel (68 nM NiCl_2_), 15 ng/ml molybdenum (156 nM MoCl_5_) or 1 μg/ml CNT-L for 8 h at 37 °C. 8-NitroG formation was examined by fluorescent immunocytochemistry as described in Methods. Hoechst, Hoechst 33258. Magnification, X200. (TIF 607 kb)

Additional file 6: Figure S5. ROS generation and oxidative DNA damage in MWCNT-treated cells. A549 cells were treated with 1 μg/ml MWCNT for indicated durations at 37 °C. (A) Flow cytometric fluorescence distributions of MWCNT-treated cells. Five μM CM-H_2_DCFDA was added 30 min before the end of the incubation. Intracellular peroxide formation was examined by flow cytometry. *Broken line*, control; *solid line*, MWCNT-treated cells. Each peak shows averaged fluorescence distribution of 3 independent samples. (B) Amount of 8-oxodG in MWCNT-treated cells. DNA was extracted from MWCNT-treated cells and 8-oxodG formation was analyzed with HPLC coupled with an ECD as described in Methods. (TIF 219 kb)

Additional file 7: Figure S6. Effects of various inhibitors on CNT-S-induced 8-nitroG formation and iNOS expression. A549 cells were treated with 1 μg/ml of CNT-S for 8 h at 37 °C in the presence of an inhibitor (1 μM 1400 W, 10 μM Bay, 1 μM CytoD, 2 mM MBCD or 50 μM MDC). Then, fluorescent immunocytochemistry was performed to detect 8-nitroG (A) and iNOS (B) as described in Methods. Hoechst, Hoechst 33258. Magnification, X200. (TIF 605 kb)

Additional file 8: Figure S7. Cytotoxic effects of iNOS and endocytosis inhibitors. A549 cells were treated with inhibitors of iNOS (1 μM 1400 W and 10 μM Bay) and endocytosis (2 mM MBCD, 50 μM MDC and 1 μM CytoD) for 8 h at 37 °C, and the cell viability was examined by MTT assay. Viability of the control cells was set at 100 %. Data represent means ± SD of 6 independent experiments. These inhibitors did not cause significant cytotoxic effects (by ANOVA followed by Tukey’s test). (TIF 582 kb)

Additional file 9: Figure S8. Inhibitory effect of TLR9 siRNA on 8-nitroG formation in MWCNT-treated HBEpC cells. HBEpC cells were transfected with 10 nM negative control siRNA or TLR9 siRNA-1 for 2 days, followed by the treatment with 1 μg/ml CNT-L for 4 h. In certain experiments, the cells were treated with siRNA alone. 8-NitroG formation was analyzed by fluorescent immunocytochemistry. Hoechst, Hoechst 33258. Magnification, X100. (TIF 342 kb)

Additional file 10: Figure S9. Cytotoxic effects of antibodies and control IgGs. A549 cells were treated with 10 μg/ml anti-HMGB1 or anti-RAGE antibody or control IgG for 16 h at 37 °C, and the cell viability was examined by MTT assay. Viability of the control cells was set at 100 %. Data represent means ± SD of 6 independent experiments. These antibodies did not cause significant cytotoxic effects (by ANOVA followed by Tukey’s test). (TIF 304 kb)

Additional file 11: Figure S10. Inhibitory effects of anti-HMGB1 and anti-RAGE antibodies on 8-nitroG formation in MWCNT-treated HBEpC cells. HBEpC cells were pretreated with 10 μg/ml anti-HMGB1 or anti-RAGE antibody for 30 min, followed by the treatment with 1 μg/ml CNT-L for 4 h. 8-NitroG formation was analyzed by fluorescent immunocytochemistry. Hoechst, Hoechst 33258. Magnification, X100. (TIF 158 kb)

Additional file 12: Figure S11. Effects of anti-TLR2 and anti-TLR4 antibodies on MWCNT-induced 8-nitroG formation. A549 cells were pretreated with 10 μg/ml anti-TLR2 or anti-TLR4 antibody for 30 min and then treated with 1 μg/ml MWCNT for 8 h. 8-NitroG formation was analyzed by fluorescent immunocytochemistry. (A) Fluorescent images of MWCNT-exposed cells pretreated with anti-TLR2 and anti-TLR4 antibodies. Hoechst, Hoechst 33258. Magnification, X200. (B) Quantitative image analysis for effects of the antibodies on 8-nitroG formation. Staining intensity per cell was analyzed by an ImageJ software. Relative staining intensity of the control was set at 1. Data represent means ± SD of 3-4 independent experiments. **p < 0.01 and ***p < 0.001, compared with the control by ANOVA followed by Tukey’s test. (TIF 604 kb)

## Abbreviations

8-nitroG: 8-nitroguanine
8-oxodG: 8-oxo-7,8-dihydro-2'-deoxyguanosine
ANOVA: analysis of variance
Bay: Bay11-7082
CM-H_2_DCFDA: 5-(and-6)-chloromethyl-2’,7’-dichlorodihydrofluorescein diacetate acetyl ester
CNT: carbon nanotube
CytoD: cytochalasin D
DMEM: Dulbecco’s modified Eagle’s medium
dsDNA: double-stranded DNA
ECD: electrochemical detector
FBS: fetal bovine serum
GSH: glutathione
HMGB1: high-mobility group box-1
HPLC: high-performance liquid chromatography
HRP: horseradish peroxidase
ICP-AES: inductively coupled plasma-atomic emission spectrometry
ICP-MS: inductively coupled plasma mass spectrometry
iNOS: inducible NO synthase
LAMP: lysosome-associated membrane protein
MBCD: methyl-β-cyclodextrin
MDC: monodansylcadaverine
MTT: 3-(4,5-dimethylthiazol-2-yl)-2,5-diphenyltetrazolium bromide
MWCNT: multi-walled CNT
NF-κB: nuclear factor-κB
NO: nitric oxide
NO_2_^-^: nitrite
NO_3_^-^: nitrate
O_2_^-^: superoxide
ONOO^-^: peroxynitrite
PBS: phosphate-buffered saline
RAGE: receptor for advanced glycation end-products
RNS: reactive nitrogen species
ROS: reactive oxygen species
siRNA: small-interfering RNA
TEM: transmission electron microscopy
TLR: Toll-like receptor
