# Supplementary material for: Multi-walled carbon nanotube induces nitrative DNA damage in human lung epithelial cells via HMGB1-RAGE interaction and Toll-like receptor 9 activation
Source: Part Fibre Toxicol. 2016 Mar 29;13:16. doi: 10.1186/s12989-016-0127-7 (PMC4812657; doi:10.1186/s12989-016-0127-7)
Supplement: Supplementary file 1 — Full metal analysis for trace elements contained in MWCNTs used in this study. (DOC 77 kb) [file 12989_2016_127_MOESM1_ESM.doc]

Table S1 Full metal analysis for trace elements contained in MWCNTs used in this study

(ND: not detected)

| Element | CNT-S  (ppm) | CNT-L  (ppm) | Detection Limit  (ppm) | Element | CNT-S  (ppm) | CNT-L  (ppm) | Detection Limit  (ppm) |
| --- | --- | --- | --- | --- | --- | --- | --- |
| Li | ND | ND | 20 | Cd | ND | ND | 10 |
| Be | ND | ND | 10 | In | ND | ND | 10 |
| B | ND | ND | 50 | Sn | ND | ND | 10 |
| Na | ND | 40 | 20 | Sb | ND | ND | 10 |
| Mg | ND | 300 | 10 | Te | ND | ND | 10 |
| Al | ND | 30 | 10 | Cs | ND | ND | 10 |
| Si | ND | ND | 5,000 | Ba | ND | ND | 10 |
| P | ND | ND | 100 | La | 20 | 10 | 10 |
| S | ND | ND | 100 | Ce | ND | ND | 10 |
| K | ND | ND | 50 | Pr | ND | ND | 10 |
| Ca | 30 | 100 | 20 | Nd | ND | ND | 10 |
| Sc | ND | ND | 10 | Sm | ND | ND | 10 |
| Ti | ND | ND | 10 | Eu | ND | ND | 10 |
| V | ND | ND | 10 | Gd | ND | ND | 10 |
| Cr | ND | 400 | 10 | Tb | ND | ND | 10 |
| Mn | ND | 30 | 10 | Dy | ND | ND | 10 |
| Fe | 50 | 2,000 | 10 | Ho | ND | ND | 10 |
| Co | 30 | 10 | 10 | Er | ND | ND | 10 |
| Ni | 4,000 | 2,000 | 10 | Tm | ND | ND | 10 |
| Cu | ND | ND | 10 | Yb | ND | ND | 10 |
| Zn | ND | ND | 100 | Lu | ND | ND | 10 |
| Ga | ND | ND | 10 | Hf | ND | ND | 10 |
| Ge | ND | ND | 10 | Ta | ND | ND | 10 |
| As | ND | ND | 10 | W | ND | ND | 10 |
| Se | ND | ND | 10 | Re | ND | ND | 10 |
| Rb | ND | ND | 10 | Os | ND | ND | 10 |
| Sr | ND | ND | 10 | Ir | ND | ND | 10 |
| Y | ND | ND | 10 | Pt | ND | ND | 20 |
| Zr | ND | ND | 10 | Au | ND | ND | 10 |
| Nb | ND | ND | 10 | Tl | ND | ND | 10 |
| Mo | 50 | 15,000 | 10 | Pb | ND | ND | 10 |
| Ru | ND | ND | 10 | Bi | ND | ND | 10 |
| Rh | ND | ND | 10 | Th | ND | ND | 10 |
| Pd | ND | ND | 10 | U | ND | ND | 10 |
| Ag | ND | ND | 10 |  |  |  |  |
