# Supplementary figures and images for: Multi-walled carbon nanotube induces nitrative DNA damage in human lung epithelial cells via HMGB1-RAGE interaction and Toll-like receptor 9 activation
Source: Part Fibre Toxicol. 2016 Mar 29;13:16. doi: 10.1186/s12989-016-0127-7 (PMC4812657; doi:10.1186/s12989-016-0127-7)

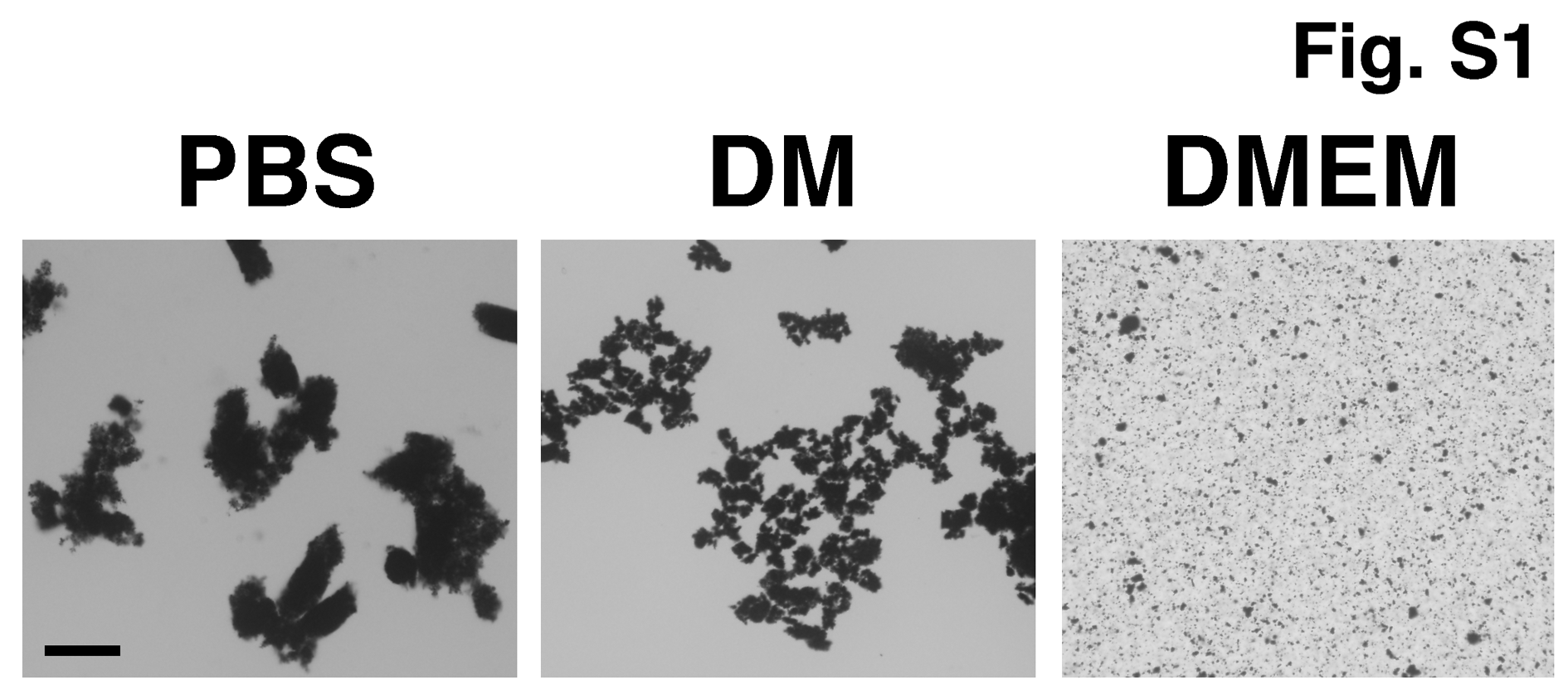

Supplement: Supplementary file 2 — Dispersion of MWCNT agglomerates in different media. CNT-L was dispersed by sonication in PBS, dispersion medium (DM) and DMEM, and then observed with a light microscope as described in Methods. Agglomerates were much more efficiently dispersed in DMEM than in PBS and dispersion medium. Bar = 50 μm. (TIF 579 kb) [file 12989_2016_127_MOESM2_ESM.tif]

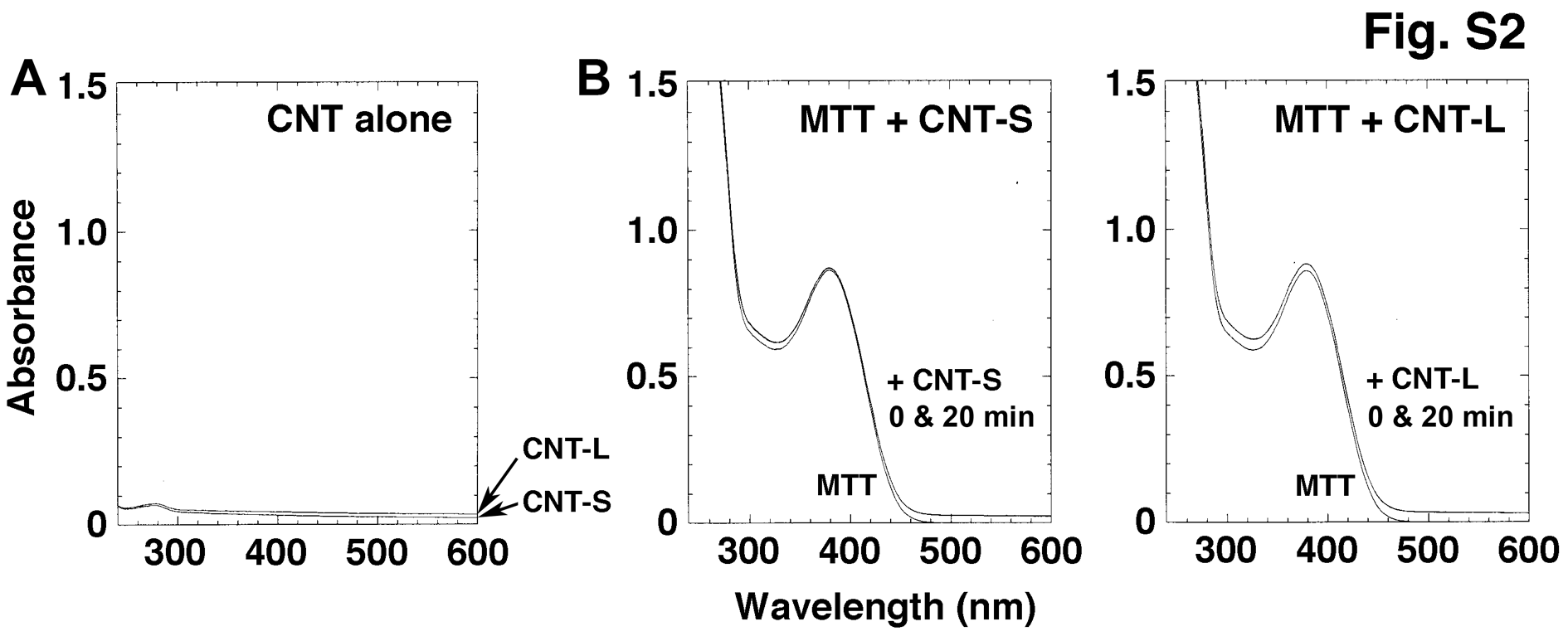

Supplement: Supplementary file 3 — Effect of MWCNT on UV-visible spectrum of MTT. (A) UV-visible spectra of MWCNT (CNT-S or CNT-L, 1 μg/ml). (B) Effect of MWCNT on the spectrum of MTT. The reaction mixture contained 0.05 mg/ml MTT, and 1 μg/ml CNT-S or CNT-L was added. The spectra were measured before (MTT) and immediately (0 min) and 20 min after the addition of MWCNT (+CNT-S and + CNT-L) at 25 °C. There was no spectral change at 0 and 20 min. (TIF 125 kb) [file 12989_2016_127_MOESM3_ESM.tif]

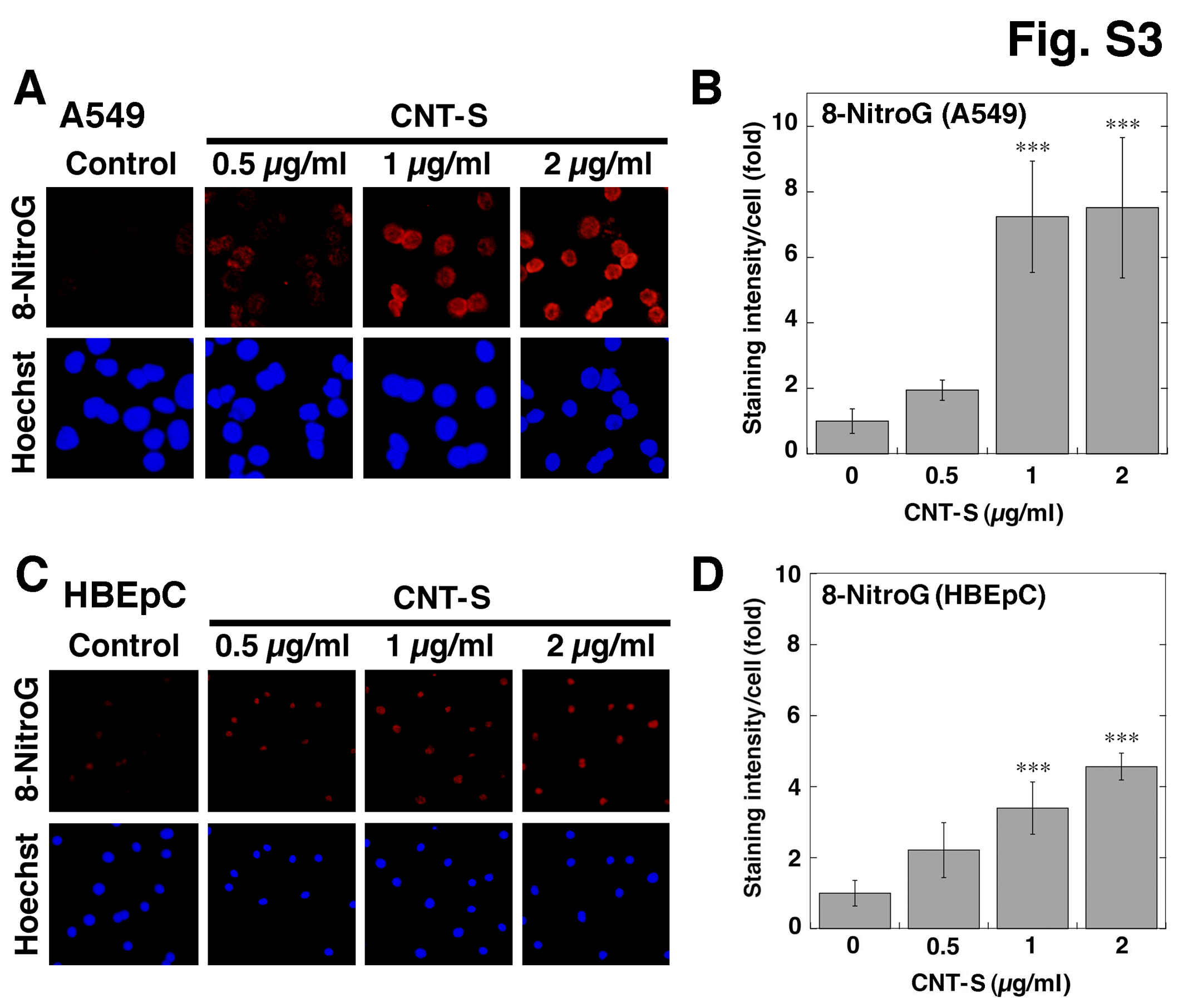

Supplement: Supplementary file 4 — DNA damage in CNT-S-treated cells. (A) Immunofluorescent images of 8-nitroG formation in CNT-S-treated A549 cells. A549 cells were incubated with CNT-S at indicated concentrations for 8 h at 37 °C, and 8-nitroG formation was examined by immunofluorescent technique as described in Methods. Hoechst, Hoechst 33258. Magnification, X200. (B) Relative staining intensity of 8-nitroG formed in CNT-S-treated A549 cells. Staining intensity per cell was analyzed by an ImageJ software. Relative staining intensity of the control was set at 1. Data represent means ± SD of 3 or 4 independent experiments. ***p < 0.001, compared with the control. (C) 8-NitroG formation in CNT-S-treated HBEpC cells. HBEpC cells were treated with CNT-S at indicated concentrations for 4 h at 37 °C, and immunofluorescent technique was performed. Magnification, X100. (D) Relative staining intensity of 8-nitroG formed in CNT-S-treated HBEpC cells. Staining intensity was analyzed as described in (B). Data represent means ± SD of 3 or 4 independent experiments. ***p < 0.001, compared with the control. Statistical analysis was performed by ANOVA followed by Tukey’s test. (TIF 654 kb) [file 12989_2016_127_MOESM4_ESM.tif]

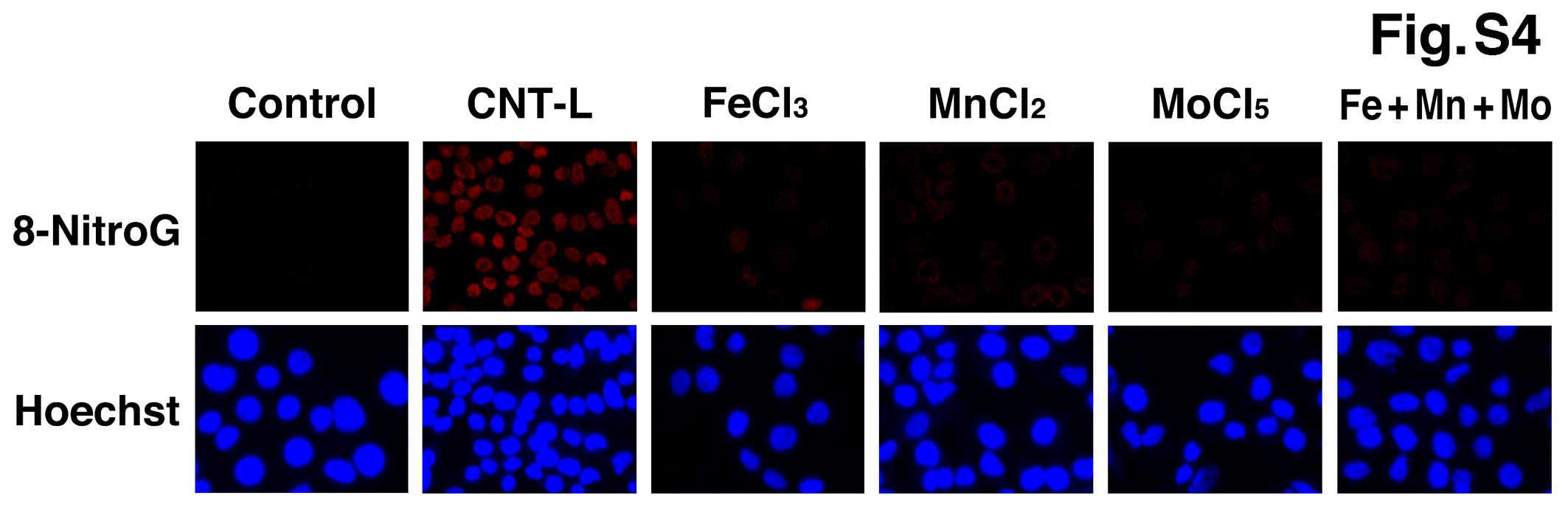

Supplement: Supplementary file 5 — Effect of trace metals contained in MWCNT on 8-nitroG formation. A549 cells were treated with 2 ng/ml iron (36 nM FeCl3), 4 ng/ml nickel (68 nM NiCl2), 15 ng/ml molybdenum (156 nM MoCl5) or 1 μg/ml CNT-L for 8 h at 37 °C. 8-NitroG formation was examined by fluorescent immunocytochemistry as described in Methods. Hoechst, Hoechst 33258. Magnification, X200. (TIF 607 kb) [file 12989_2016_127_MOESM5_ESM.tif]

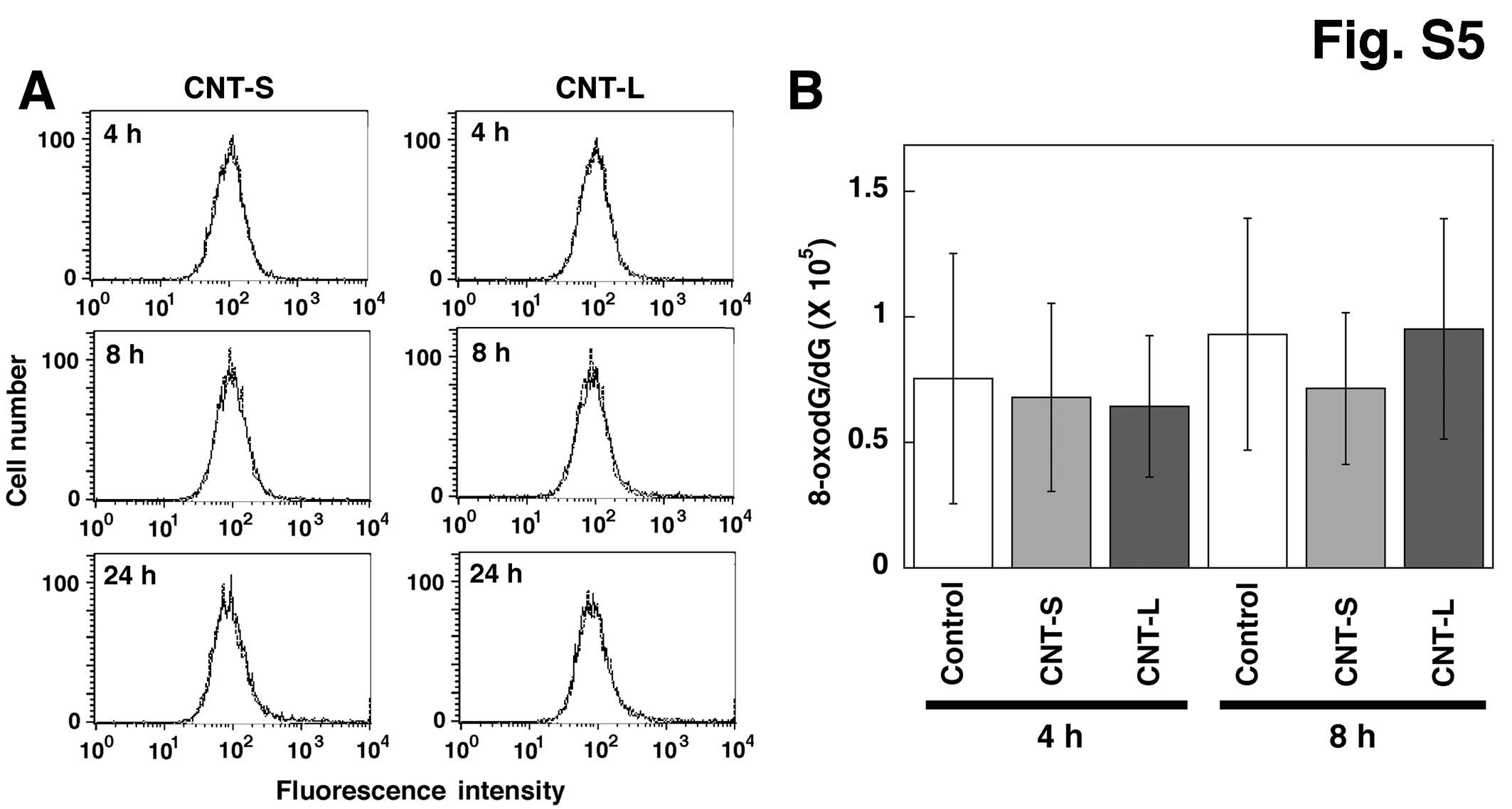

Supplement: Supplementary file 6 — ROS generation and oxidative DNA damage in MWCNT-treated cells. A549 cells were treated with 1 μg/ml MWCNT for indicated durations at 37 °C. (A) Flow cytometric fluorescence distributions of MWCNT-treated cells. Five μM CM-H2DCFDA was added 30 min before the end of the incubation. Intracellular peroxide formation was examined by flow cytometry. Broken line, control; solid line, MWCNT-treated cells. Each peak shows averaged fluorescence distribution of 3 independent samples. (B) Amount of 8-oxodG in MWCNT-treated cells. DNA was extracted from MWCNT-treated cells and 8-oxodG formation was analyzed with HPLC coupled with an ECD as described in Methods. (TIF 219 kb) [file 12989_2016_127_MOESM6_ESM.tif]

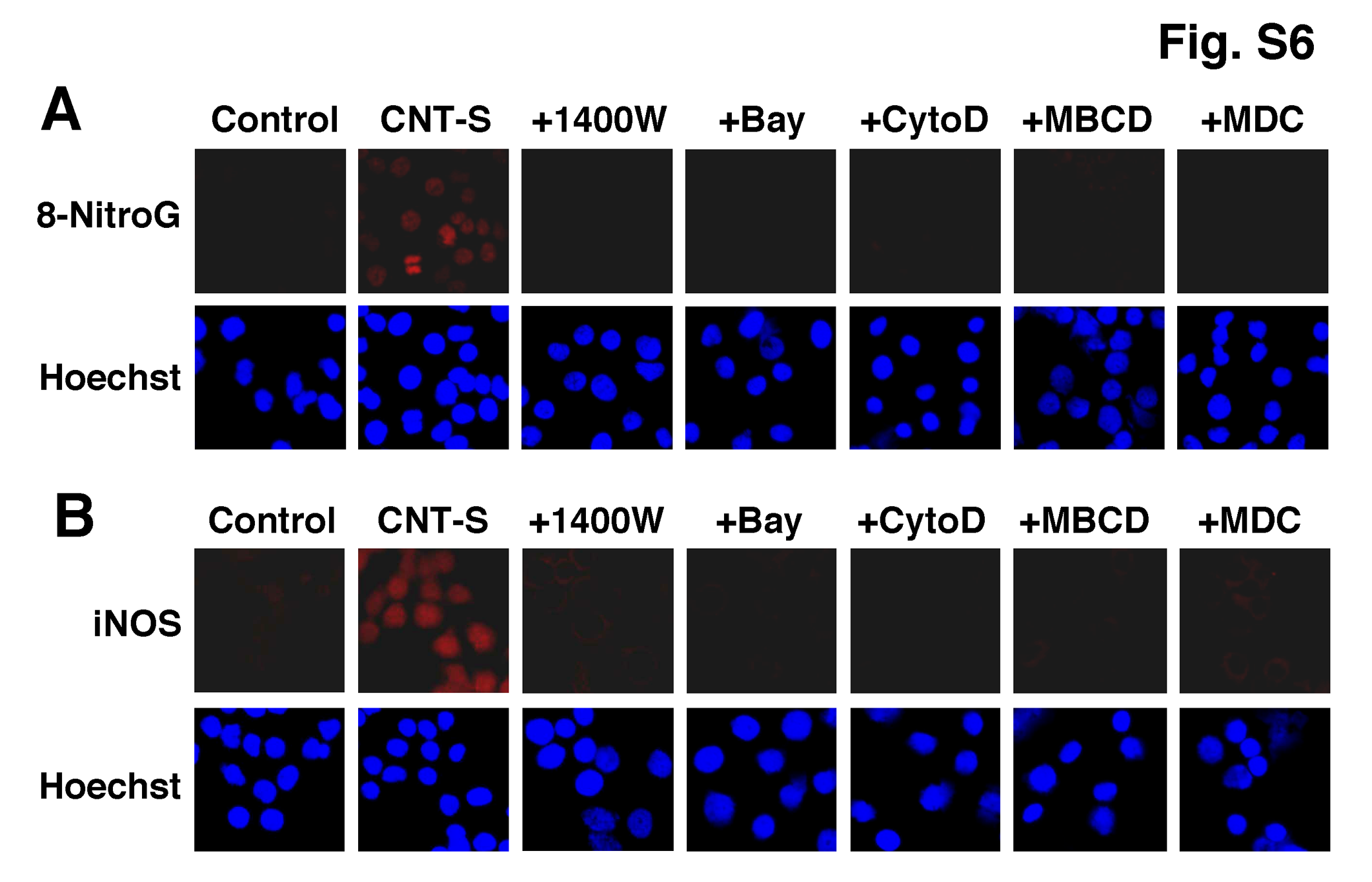

Supplement: Supplementary file 7 — Effects of various inhibitors on CNT-S-induced 8-nitroG formation and iNOS expression. A549 cells were treated with 1 μg/ml of CNT-S for 8 h at 37 °C in the presence of an inhibitor (1 μM 1400 W, 10 μM Bay, 1 μM CytoD, 2 mM MBCD or 50 μM MDC). Then, fluorescent immunocytochemistry was performed to detect 8-nitroG (A) and iNOS (B) as described in Methods. Hoechst, Hoechst 33258. Magnification, X200. (TIF 605 kb) [file 12989_2016_127_MOESM7_ESM.tif]

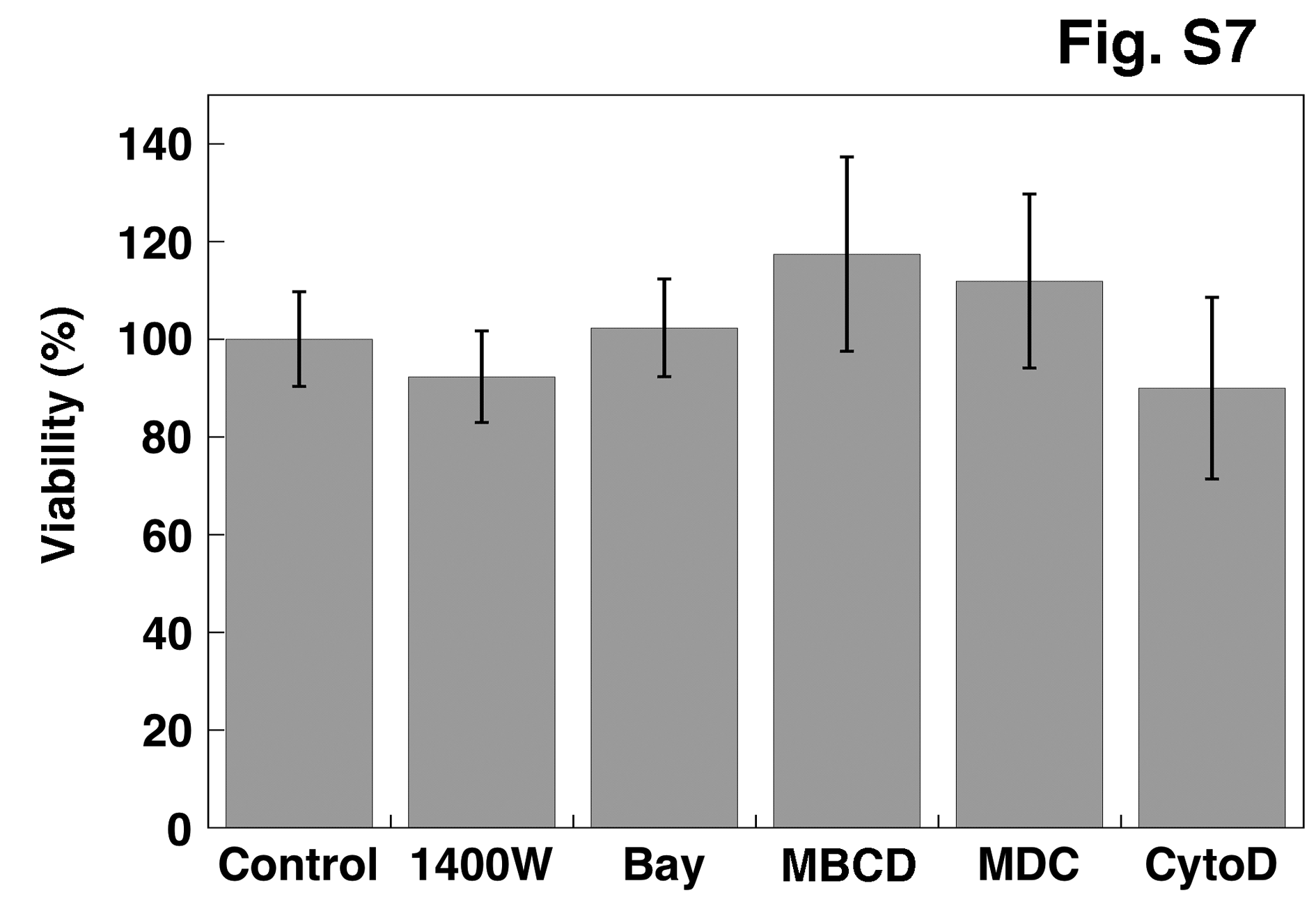

Supplement: Supplementary file 8 — Cytotoxic effects of iNOS and endocytosis inhibitors. A549 cells were treated with inhibitors of iNOS (1 μM 1400 W and 10 μM Bay) and endocytosis (2 mM MBCD, 50 μM MDC and 1 μM CytoD) for 8 h at 37 °C, and the cell viability was examined by MTT assay. Viability of the control cells was set at 100 %. Data represent means ± SD of 6 independent experiments. These inhibitors did not cause significant cytotoxic effects (by ANOVA followed by Tukey’s test). (TIF 582 kb) [file 12989_2016_127_MOESM8_ESM.tif]

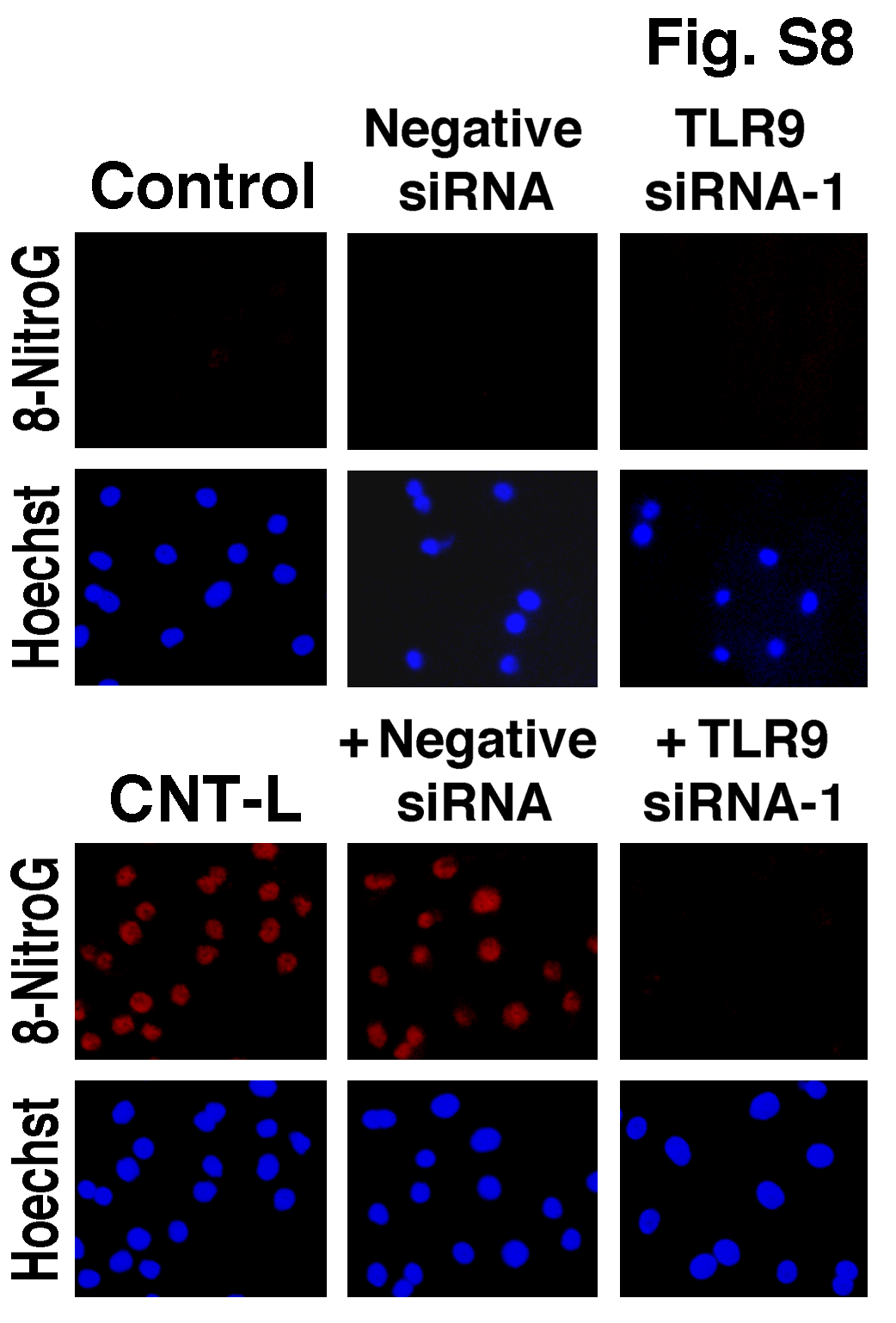

Supplement: Supplementary file 9 — Inhibitory effect of TLR9 siRNA on 8-nitroG formation in MWCNT-treated HBEpC cells. HBEpC cells were transfected with 10 nM negative control siRNA or TLR9 siRNA-1 for 2 days, followed by the treatment with 1 μg/ml CNT-L for 4 h. In certain experiments, the cells were treated with siRNA alone. 8-NitroG formation was analyzed by fluorescent immunocytochemistry. Hoechst, Hoechst 33258. Magnification, X100. (TIF 342 kb) [file 12989_2016_127_MOESM9_ESM.tif]

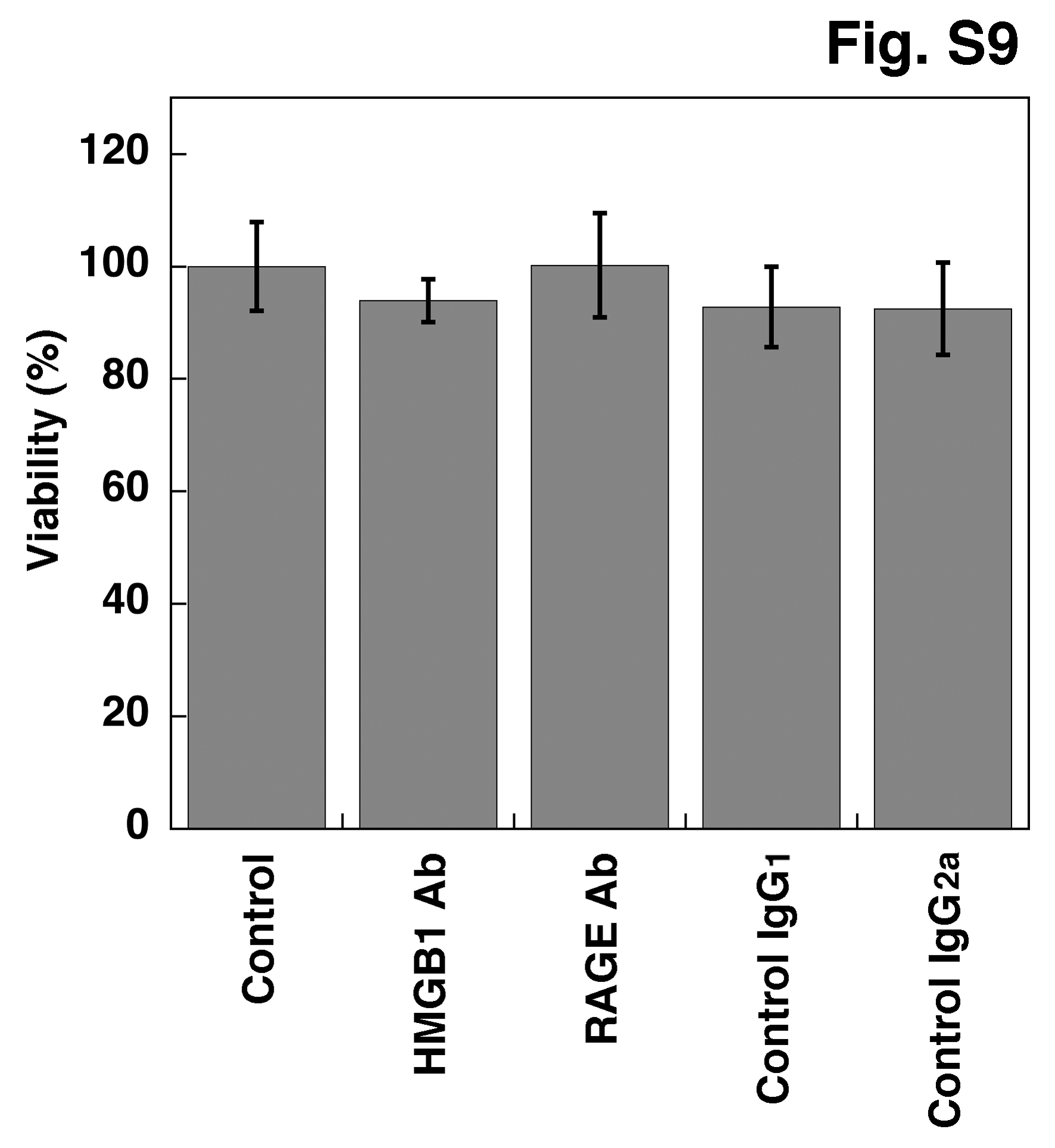

Supplement: Supplementary file 10 — Cytotoxic effects of antibodies and control IgGs. A549 cells were treated with 10 μg/ml anti-HMGB1 or anti-RAGE antibody or control IgG for 16 h at 37 °C, and the cell viability was examined by MTT assay. Viability of the control cells was set at 100 %. Data represent means ± SD of 6 independent experiments. These antibodies did not cause significant cytotoxic effects (by ANOVA followed by Tukey’s test). (TIF 304 kb) [file 12989_2016_127_MOESM10_ESM.tif]

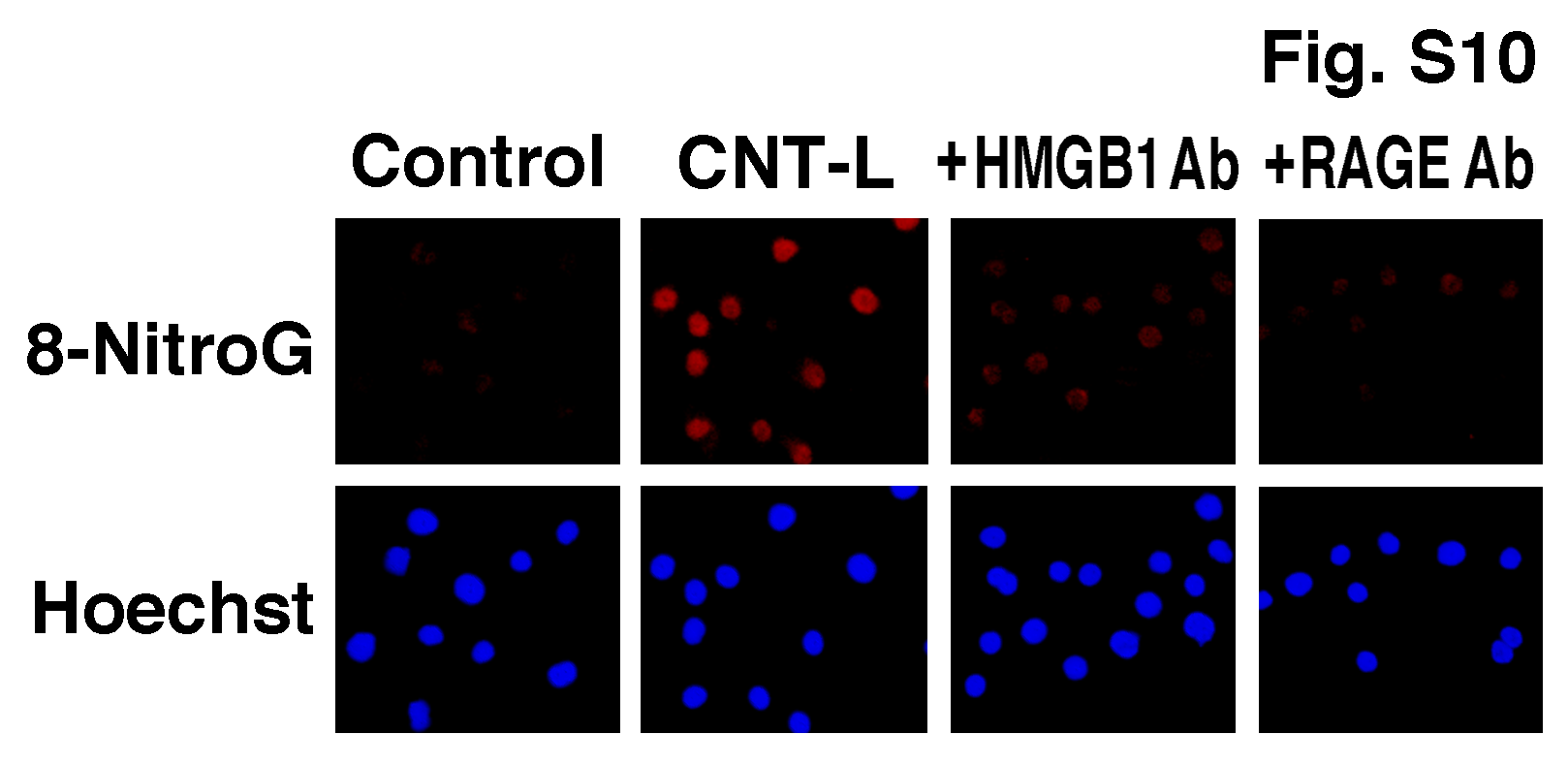

Supplement: Supplementary file 11 — Inhibitory effects of anti-HMGB1 and anti-RAGE antibodies on 8-nitroG formation in MWCNT-treated HBEpC cells. HBEpC cells were pretreated with 10 μg/ml anti-HMGB1 or anti-RAGE antibody for 30 min, followed by the treatment with 1 μg/ml CNT-L for 4 h. 8-NitroG formation was analyzed by fluorescent immunocytochemistry. Hoechst, Hoechst 33258. Magnification, X100. (TIF 158 kb) [file 12989_2016_127_MOESM11_ESM.tif]

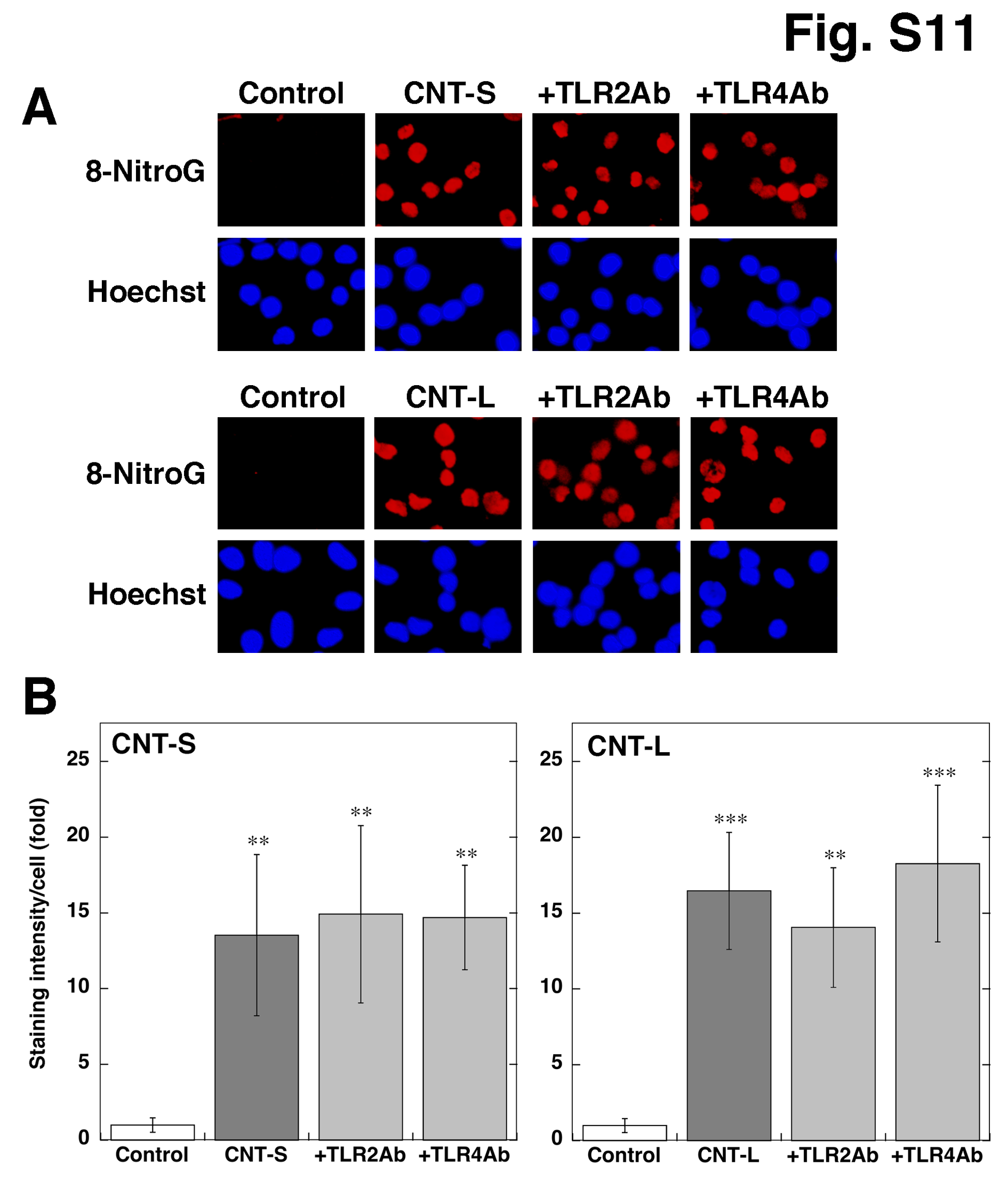

Supplement: Supplementary file 12 — Effects of anti-TLR2 and anti-TLR4 antibodies on MWCNT-induced 8-nitroG formation. A549 cells were pretreated with 10 μg/ml anti-TLR2 or anti-TLR4 antibody for 30 min and then treated with 1 μg/ml MWCNT for 8 h. 8-NitroG formation was analyzed by fluorescent immunocytochemistry. (A) Fluorescent images of MWCNT-exposed cells pretreated with anti-TLR2 and anti-TLR4 antibodies. Hoechst, Hoechst 33258. Magnification, X200. (B) Quantitative image analysis for effects of the antibodies on 8-nitroG formation. Staining intensity per cell was analyzed by an ImageJ software. Relative staining intensity of the control was set at 1. Data represent means ± SD of 3-4 independent experiments. **p < 0.01 and ***p < 0.001, compared with the control by ANOVA followed by Tukey’s test. (TIF 604 kb) [file 12989_2016_127_MOESM12_ESM.tif]
